# Docking cholesterol to integral membrane proteins with Rosetta

**DOI:** 10.1371/journal.pcbi.1010947

**Published:** 2023-03-27

**Authors:** Brennica Marlow, Georg Kuenze, Jens Meiler, Julia Koehler Leman

**Affiliations:** 1 Center for Structural Biology, Vanderbilt University, Nashville, Tennessee, United States of America; 2 Chemical and Physical Biology Program, Vanderbilt University, Nashville, Tennessee, United States of America; 3 Department of Chemistry, Vanderbilt University, Nashville, Tennessee, United States of America; 4 Institute for Drug Discovery, Leipzig University Medical School, Leipzig, Germany; 5 Center for Computational Biology, Flatiron Institute, Simons Foundation, New York, New York, United States of America; Tel Aviv University, ISRAEL

## Abstract

Lipid molecules such as cholesterol interact with the surface of integral membrane proteins (IMP) in a mode different from drug-like molecules in a protein binding pocket. These differences are due to the lipid molecule’s shape, the membrane’s hydrophobic environment, and the lipid’s orientation in the membrane. We can use the recent increase in experimental structures in complex with cholesterol to understand protein-cholesterol interactions. We developed the RosettaCholesterol protocol consisting of (1) a prediction phase using an energy grid to sample and score native-like binding poses and (2) a specificity filter to calculate the likelihood that a cholesterol interaction site may be specific. We used a multi-pronged benchmark (self-dock, flip-dock, cross-dock, and global-dock) of protein-cholesterol complexes to validate our method. RosettaCholesterol improved sampling and scoring of native poses over the standard RosettaLigand baseline method in 91% of cases and performs better regardless of benchmark complexity. On the β2AR, our method found one likely-specific site, which is described in the literature. The RosettaCholesterol protocol quantifies cholesterol binding site specificity. Our approach provides a starting point for high-throughput modeling and prediction of cholesterol binding sites for further experimental validation.

## Author summary

Over 30% of the human proteome consists of integral membrane proteins. These proteins are critical in many biological processes, implicated in many diseases, and targeted by up to 50% of pharmaceutical drugs. The lipids of the membrane environment affect the function of integral membrane proteins. Protein-lipid interactions range from specific interactions at particular sites on the protein to nonspecific interactions involving changes to the physical membrane properties. It has become increasingly clear that cholesterol is one of the major proponents affecting integral membrane protein function. To study integral membrane protein-cholesterol interactions, we developed a docking algorithm in the Rosetta software, including a score that quantifies likely-specific interaction sites.

This is a *PLOS Computational Biology* Methods paper.

## Introduction

Integral membrane proteins (IMP) are central to various fundamental biological processes and constitute ~30% of the human proteome [1]. They carry out diverse functions such as intracellular adhesion, enzymatic activity, transport, cell-to-cell recognition, and signal transduction. Numerous diseases are linked to malfunctions in IMPs, and ~50% of pharmaceutical drugs target them [2]. Processes such as signal transduction and selective transport of molecules performed in the cellular membrane were solely attributed to IMPs. However, it is now widely recognized that lipids alter IMP structure, dynamics, and function and vice versa [3]. Lipids have a significant degree of diversity in their structures due to the modifications of their polar head groups and hydrophobic tails. Lipid-protein interactions include specific lipid binding to the protein or nonspecific interactions by altering the properties of the membrane in which the protein resides. It is difficult to characterize these interactions separately because the likelihood of random interactions between the protein and lipids is prevalent due to the high ratio of lipids to protein in the plasma membrane, being 50:1 [4,5] Sterols are a distinct class of lipids that are defined by their tetracyclic structure. The sterol cholesterol is a vital component of eukaryotic cell membranes, making up ~30% of their composition and regulating its physical properties [6–8]. Cholesterol has been shown to be a crucial modulator of many IMPs. For example, cholesterol in the brain diminishes cannabinoid affinity towards the Cannabinoid 1 (CB1) receptor [9]. The cholesterol-binding site on CB1 is suggested to be an allosteric modulator that disrupts receptor activation by CB1 agonists.

Both experimental and computational approaches are used to study different forms of protein-cholesterol interactions. Some experimental techniques to identify cholesterol binding sites are structural characterization (X-ray, cryoEM, NMR), cholesterol content manipulation [10], and fluorescent labeling of cholesterol analogs [11,12]. Structural characterization techniques have allowed the visualization of lipids in contact with the protein surface. However, the introduction of stabilizing mutations is sometimes required for the crystallization process, which can significantly affect the protein’s function. Cholesterol content manipulation only directly explains nonspecific interactions. Lastly, with fluorescence experiments, due to the small size of cholesterol and its involvement in the surrounding membrane environment, it is difficult to find a cholesterol fluorophore with the same features as cholesterol. Due to this, much of the results are measured indirectly [13]. Due to the insolubility of cholesterol in aqueous solvents, investigating protein-cholesterol interactions can be difficult, motivating the use of computational techniques.

Nonspecific and specific protein-cholesterol interactions can be studied with molecular dynamics (MD) simulations using complex lipid compositions, which include structural flexibility and entropic effects. Specific interactions can also be probed with protein-ligand docking tools that aim to predict the binding orientations of a ligand in a receptor-binding site. Many docking tools are available, such as RosettaLigand [14], AutoDock [15–17], and Glide [18–20] each differing in their sampling and scoring algorithm. These algorithms are built to dock ligands into the water-exposed central cavity of proteins. They allow for *virtual* high throughput screening (vHTS) or modeling of single-point mutations *in silico*. For example, RosettaLigand can screen medium sized libraries with all-atom representation and flexibility of the protein and ligand [21].

However, the prediction of protein-lipid interactions is not usually the application of docking tools, so they lack optimized protocols to dock lipids to IMPs. To our knowledge, protein-lipid docking protocols have only been developed by Profacgen (unavailable to the public) [22]. However, in the specific case of cholesterol, Profacgen doesn’t try to discriminate between specific and nonspecific protein-cholesterol interactions.

In this work, we model protein-cholesterol interactions using a new protocol named RosettaCholesterol (RC). The protocol uses a novel scoring grid and a two-prong specificity filter. The current RosettaLigand energy grid fails to consider the specifics of lipids, e.g., their orientation preference in the membrane and low structure order. So, we introduce the LipidMemGrid energy grid that accounts for lipid directionality and increases the sampling of native-like protein-lipid conformations. The specificity filter uses both structural and evolutionary constraints [23–25] to predict possible cholesterol binding-site specificity by ranking and scoring realistic models.

## Results and discussion

The RC protocol builds on the current RosettaLigand [14] algorithm to adapt the sampling and scoring function to model protein-cholesterol interactions. The RC protocol utilizes a new scoring grid called LipidMemGrid and a two-prong filter that calculates the specificity score of a cholesterol binding site. The LipidMemGrid scoring grid generally characterizes a ligand as a lipid by using the structural properties of the lipid to `correctly`orient it at the interface of the membrane and a protein and accurately score those interactions. The two-step filter in the RC protocol explicitly addresses whether a proposed binding pocket is likely to be specific. We use a combination of structural and evolutionary properties of each docked binding site to determine the likelihood of them being specific. The docking and scoring strategies are detailed in the Methods section.

### 1. RC is a scoring method for modeling protein-cholesterol interactions

**Fig 1:**
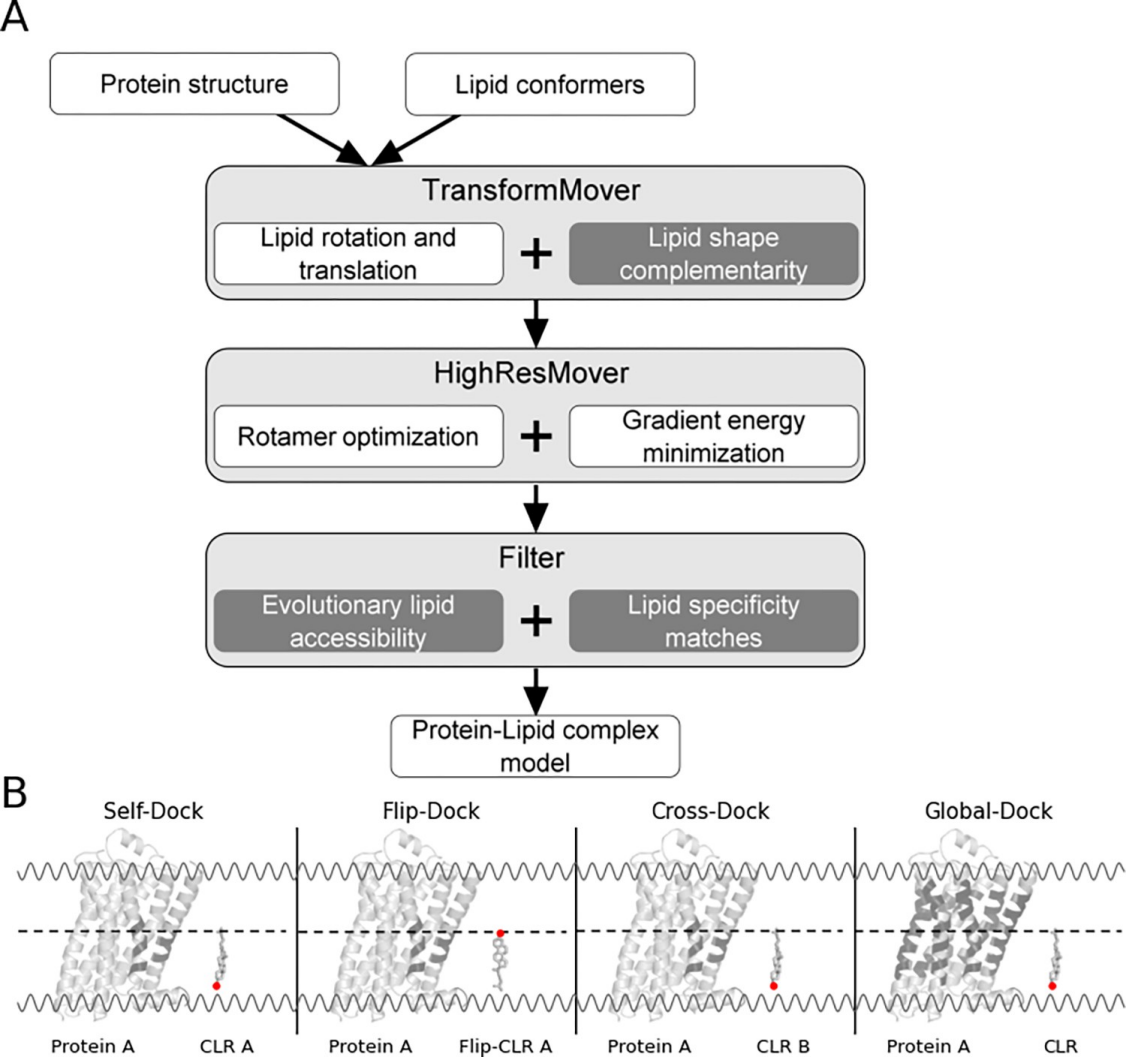
Schematic of the RC protocol. (A) The protocol is divided into docking and filter sections. During the docking (or prediction phase) we use centroid (TransformMover) and full-atom (HighResMover) representations of the protein structure and lipid conformers. Protein and lipid conformer moves are repeated based on the accept/reject Metropolis Monte Carlo ratio. In the filtering section, the evolutionary lipid accessibility and lipid specificity matches are used to determine the specificity score. The specificity score determines the probability of a protein-cholesterol binding site being specific. The output is an individual protein-lipid complex and a csv file. Highlighted in gray are the additional steps added to the current RosettaLigand protocol that compose the RC protocol. (B) Cartoon representation of each benchmark setup. Protein A and CLR A (self-dock); Protein A and Flip-CLR A (flip-dock); Protein A and CLR B (cross-dock); Protein A and CLR (global-dock). A dashed line depicts the center of the membrane. Protein A is shown in cartoon representation, while CLR is shown in stick representation. An example of the protein area cholesterol is docked into for each benchmark is colored black.

The RC protocol (Fig 1A) uses the RosettaMP membrane framework [26] with complete ligand and protein flexibility to investigate the specificity of protein-cholesterol interactions. RC is accomplished in three steps: the centroid-based TransformMover, the full-atom HighResMover, and the specificity filter (Fig 1A). In the TransformMover, the cholesterol molecule is translated and rotated randomly on the interface of the IMPs with the implicit membrane. After each move, scores are calculated using the LipidMemGrid, a complementary energy grid (including van der Waals interactions). The grid is generated around cholesterol with specific attenuation to the 3β-OH group at the head and the atoms forming the tail. For other lipid molecules, the user can define the head and tail atoms of the lipid. For example, the phosphatidylcholine lipid head atom would be the phosphorus atom, and the tail atoms would be C15 (on the palmityl acyl chain) and C17 (on the oleic acyl chain). In this work, the head atom is the O1 atom in cholesterol, and the tail atom is C25. The head and tail atom scores are weighted higher than the whole molecule scores to ensure the correct directionality of cholesterol in the membrane environment. The lowest scoring Pose is passed on to the HighResMover.

The HighResMover samples and repacks side-chain rotamers and implements small perturbations to the cholesterol molecule, optimizing the protein-cholesterol interface. We score the lipid-membrane interface using the *franklin2019* [27] implicit membrane score function. Scores are accepted/rejected based on the Metropolis Monte Carlo criterion.

In the filtering step, we provide a specificity likelihood score. As the protein-cholesterol binding Poses are optimized, we independently compute the rate of evolution of the binding site compared to other lipid-accessible residues and lipid specificity fingerprints. We then combine these two independent terms into a weighted sum called the specificity score (see Methods).

The main focus of this work is to model protein-cholesterol complexes and show that the RC protocol can be used to predict specificity likelihoods. We evaluate the protocol in two parts: prediction (using the TransformMover and HighResMover) and the complete RC protocol (using the TransformMover, HighResMover, and specificity filter)

A three-part benchmark strategy is used to evaluate the RC prediction phase performance and show its improvement over standard RosettaLigand (Fig 1B). (1) A self-docking benchmark was performed to evaluate which docking protocol better recapitulates the crystal structure binding mode. Each cholesterol is docked into the IMP it was co-crystallized with, including full protein and ligand flexibility. (2) A flip-docking benchmark was performed to evaluate which docking method can recognize cholesterol as a lipid and correctly orient its head and tail atoms in the membrane. Cholesterol is rotated 180 degrees about the membrane plane and is docked into its co-crystallized IMP. (3) The cross-docking benchmark is used to evaluate which docking protocol better demonstrates cholesterol recognition of different IMPs. Cholesterol from one protein-ligand complex structure is docked into a protein from a different co-crystallized protein-cholesterol complex, and vice versa.

Lastly, we conduct a global docking experiment to evaluate the complete RC protocol. Cholesterol is randomly docked over the lipid-accessible regions of the IMP using a Monte Carlo Metropolis search. We determine its ability to find cholesterol binding sites *de novo* and rank these according to their possible specificity.

### 2. RC improves docking of native-like binding Poses

Here, our goal is to assess the ability of the RC protocol to differentiate between sampling native-like Poses from non-native Poses. We compare the outputs of RC, RosettaLigand, and AutoDock protocols to each other in the self-dock, flip-dock, and cross-dock benchmarks.

**Fig 2:**
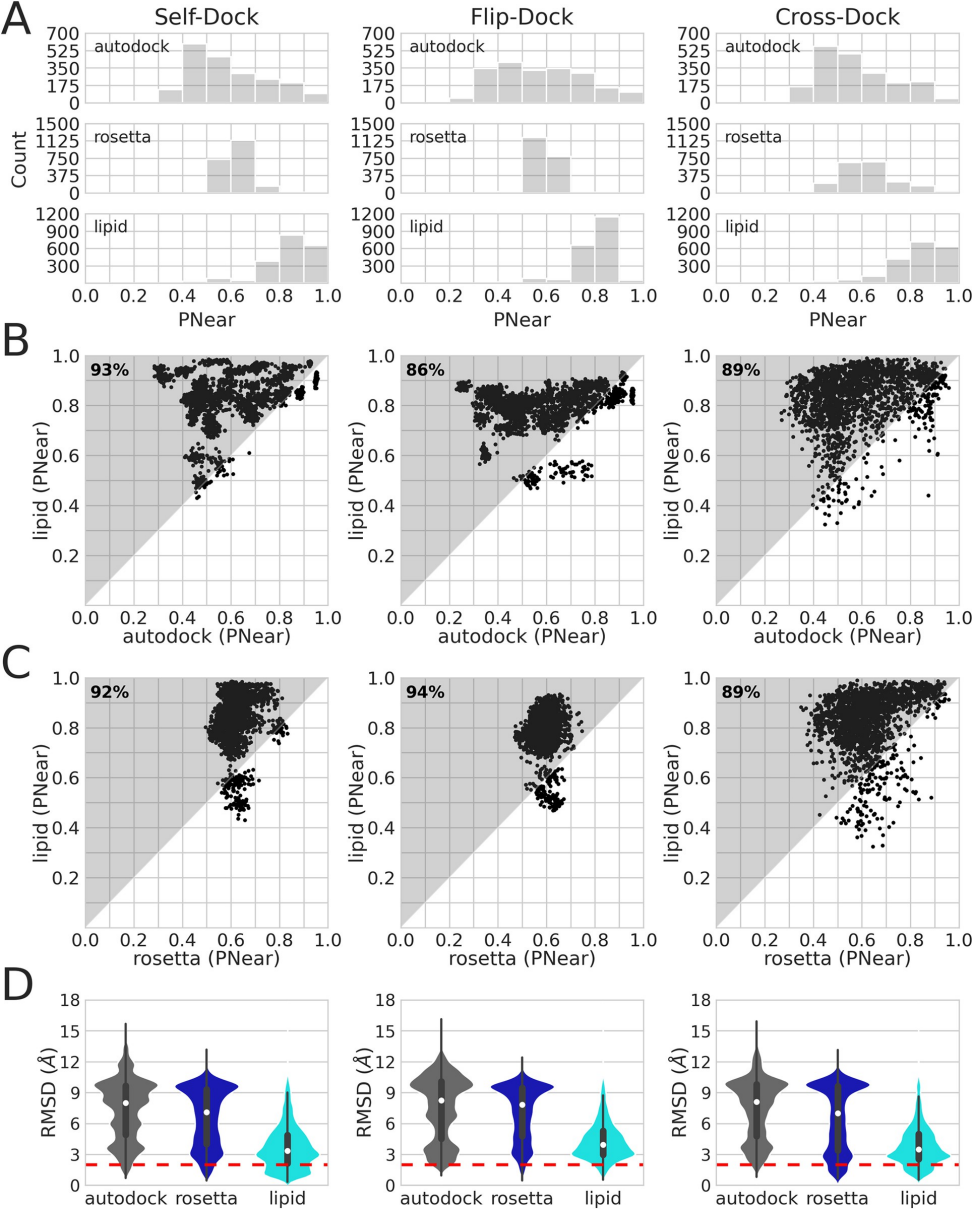
Comparing protein-cholesterol binding site `nativeness`in the self-dock, flip-dock, and cross-dock benchmarks. (A) Histogram of PNear values for each benchmark. PNear values range from 0 (non-native) to 1 (native). The docking protocol is labeled in the upper-left corner of each plot. (B-C) Scatter plot of the PNear values of the three different methods: RC (lipid) versus AutoDock and RC (lipid) versus RosettaLigand. Improved RC PNear values are highlighted in gray and quantified in the upper-left corner of each plot. (D) Distribution of RMSDs. RMSD values range from 0 to 18 Å. AutoDock models are gray, RosettaLigand models are blue, and RC (lipid) models are cyan. The red dotted lines mark 2 Å.

Firstly, we evaluate each protocol’s ability to converge on a low-scoring protein-cholesterol model with a deep energy gap that separates the best model from other similar models using the metric PNear, described in [28,29] as a measure of the "funnel-likeness" of a score versus RMSD plot. This metric ranges from 0 to 1 and is derived from a Boltzmann representation of a system and is used to calculate the density of converged low-energy models with respect to the defined reference state. We define the reference state as the experimental structure from the Orientations of Proteins in Membranes (OPM) database [30]. PNear close to 0 suggests there is little convergence towards the defined reference state and therefore is unlikely to be the observed state, whereas values close to 1 indicate an energetic convergence onto the low-energy near-native Pose. Since our goal is to capture convergence towards a low energy conformation, higher Pnear values are desired. RC produces more native-like models than AutoDock and RosettaLigand (Fig 2A): The median PNear across the three benchmarks is 0.84 for RC, 0.56 for AutoDock, and 0.61 for RosettaLigand.

$$PNear=\frac{\sum_{i=1}^{N}e^{\frac{{-RMSD}_{i}^{2}}{\lambda^{2}}}e^{-\frac{E_{i}}{k_{B}T}}}{\sum_{j=1}^{N}e^{-\frac{E_{j}}{k_{B}T}}}$$

where λ = 1.5 and kBT = 0.62.

Additionally, we directly compare the RC (y-axis) to AutoDock and RosettaLigand results (x-axes in Fig 2B and 2C). Data points in the upper triangle highlighted in gray denote an improvement of RC over AutoDock or RosettaLigand, whereas data points in the lower triangle in white denote a worse performance (as indicated by PNear). We quantify the percentage of data points in the gray triangle (upper left corner) to show the improvement of RC over AutoDock and RosettaLigand.

RC produces more low-energy near-native models as compared to AutoDock and RosettaLigand. Of the 202,500 models generated, RC showed improvement over AutoDock for 93% of the models for self-dock, 86% for flip-dock, and 89% for cross-dock. The multiple populations in the self-dock and flip-dock benchmarks (Fig 2B) are likely due to AutoDock’s clustering method. These clusters disappear in the cross-dock benchmark.

RC improves over RosettaLigand for 92% of the models for self-dock, 94% for flip-dock, and 89% for cross-dock (Fig 2C). There is a population of models in which RC cannot recapitulate the low-energy near-native models in the self-dock and the flip-dock benchmarks. They belong to the PDBs 6IYC (APP gamma-secretase complex), 6OMM (GPCR formylpeptide receptor), and 6UY0 (MRP1 ABC transporter). For these three examples, the RosettaLigand median PNear is 0.63 (compared to 0.55 for RC), showing that RosettaLigand only leads to a marginal improvement compared to RC. For PDB 6IYC the binding site mostly includes loops which are inherently more flexible than the rest of the alpha-helical protein structure. In the RC protocol, protein flexibility is simulated in the high-resolution stage. It may be difficult for RC to find a low-energy conformation like the native with such large degrees of freedom in the binding site due to the loops. For PDB 6OMM, the protein has a small surface area (216 Å^2^) for cholesterol to bind to. In our dataset, we rarely found binding sites with surface areas below 219 Å^2^ (12%). Lastly, in PDB 6UY0, the cholesterol molecule is in a horizontal (perpendicular to the membrane normal) conformation. RC is biased toward the vertical (parallel to the membrane normal) conformation, making it difficult to recapitulate a horizontal conformation. We note that we only see this behavior in the self-dock and flip-dock cases. In the cross-docking experiments, there was no consensus on the failures. The binding sites’ global shape varies for different proteins, and their side chain residues have different orientations. The self-dock and flip-dock benchmarks may overfit for the ideal Pose in the crystal structure, and never sample other low-energy conformations. With cross-docking, RC may sample more conformations finding other low-energy wells.

Finally, we show that the RMSD distribution confirms that RC produces more native models than AutoDock and RosettaLigand (Fig 2D). Native models are defined as models with a ligand root mean square deviation (RMSD) of 2 Å or less when compared to their experimental structure. The higher density at low RMSDs indicates success at sampling native conformations. On average, RC produces five times the number of models that AutoDock or RosettaLigand produces with RMSDs < = 2Å. RosettaLigand samples two clusters where the oxygen atom points into and out of the membrane, corresponding to RMSDs < = 3.5Å and > = 9.5Å. In the majority of cases (62%), RosettaLigand samples two populations even when cholesterol starts in the native position at the beginning of the protocol, whereas RC eliminates the non-native conformation during sampling (13%). In the AutoDock models, we observe polymodal distributions that highlight non-biologically relevant conformations (Fig A in S1 Text).

RosettaLigand samples a bimodal distribution because the scoring grid used in the TransformMover does not account for cholesterol orientation in the membrane. In contrast, expected biologically irrelevant conformations (i.e. the head group pointing into the membrane core) are removed when using the LipidMemGrid in the TransformMover.

### 3. The RC protocol recapitulates the native binding site tilt angle of cholesterol

**Fig 3:**
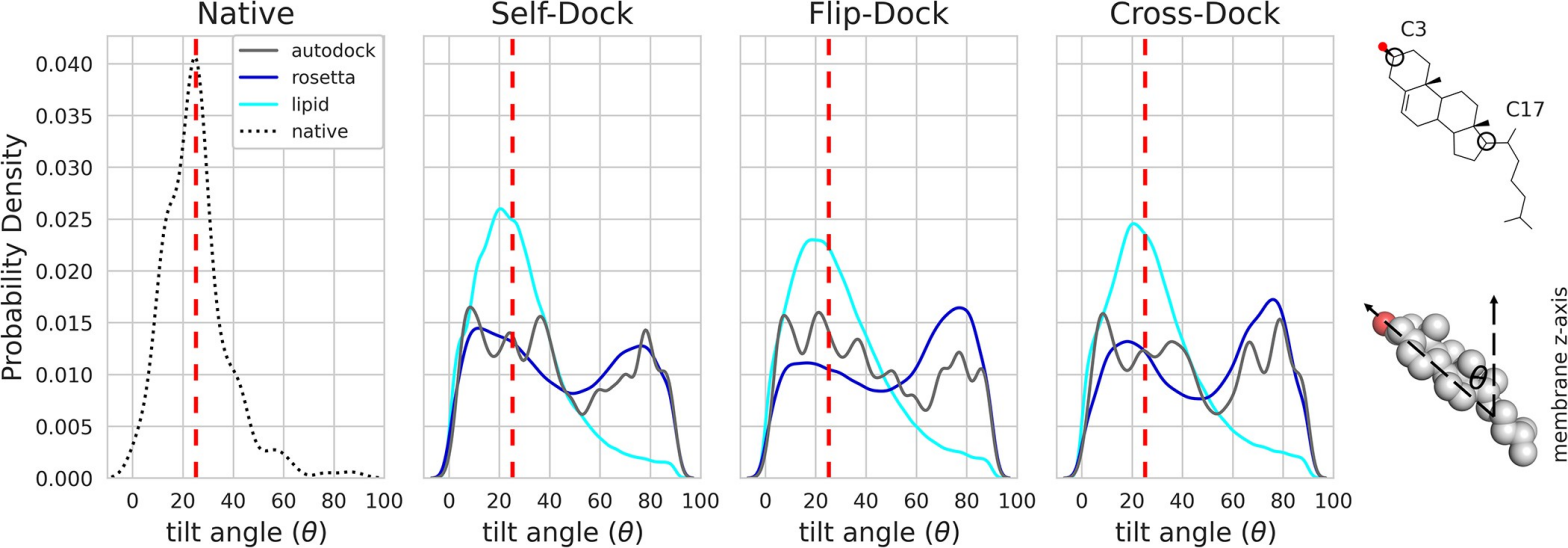
Cholesterol tilt angle in the self-dock, flip-dock, and cross-dock benchmarks. The tilt angle θ is defined as the angle between the sterol plane (C3 and C17 atoms) and the membrane normal (z axis). A probability density quantifies the tilt angle distribution. AutoDock is shown in gray, RosettaLigand in blue, and RC (lipid) in cyan. The tilt angle distribution found for cholesterol in experimental X-ray and cryo-EM structures is shown as a black dotted line. The red dotted line marks the average tilt angle of the native structure (25 degrees).

Cholesterol affects the structural and mechanical properties of the lipid membrane, such as its rigidity [31]. The tilt angle as a metric for cholesterol’s packing in the membrane is one parameter to observe. We define the tilt angle θ of cholesterol as the angle between the cholesterol plane vector (connecting cholesterol C3 and C17 atoms) and the membrane normal. Research suggests that the cholesterol tilt angle is concentration-dependent: At 1 mol% concentration, the average tilt angle is ~60 degrees, where the hydroxyl group is pointed at the membrane surface; at 20 mol%, the average tilt angle is between ~14–19 degrees [32,33]. In our set of native structures used for deriving the LipidMemGrid, the average tilt angle is 25 degrees (red dotted line in Fig 3).

Here we determine the probability densities of the cholesterol tilt angle in protein-cholesterol interfaces in models produced by RC, AutoDock, and RosettaLigand protocols. We plot the tilt angle between 0 and 100 degrees and denote the average tilt angle of the LipidMemGrid derivation set as a red dashed line. The tilt angle of the binding site in RC models is similar to that of the native structures. The tilt angle distribution of RC models is unimodal, with a population at 29 degrees in all three benchmarks. In RosettaLigand models, we observed two distinct clusters equally distributed in all three benchmarks (with averages of 23 and 71 degrees). A polymodal distribution is observed in AutoDock models.

The RosettaLigand population at 71 degrees represents cholesterol becoming closer to aligning perpendicular (90 degrees) to the membrane normal. i.e. the membrane plane. Some studies have suggested that cholesterol at certain concentrations in poly-unsaturated membranes may adopt a conformation perpendicular to the membrane normal [34]. Cholesterol in the PDB structure 6UY0 (in the test set) is in such a perpendicular conformation. In the 80–100 degree range, RC could recapitulate the perpendicular conformation of 6UY0 at 25%. AutoDock and RosettaLigand recapitulate the 6UY0 perpendicular conformation at 42% and 24%, respectively (see Fig B in S1 Text).

The three algorithms (AutoDock, RosettaLigand, RC) all have populations similar to the average tilt angle of our derivation set, suggesting favorable orientations. However, only RC shows a unimodal distribution that allows distinguishing native-like from non-native binding orientations. These results show that a native-like cholesterol binding site in an IMP requires an orientation of cholesterol with respect to the membrane normal in a low free energy conformation. Even if the protein-cholesterol complex model has a favorable interface score, it is less likely to be a native binding site if cholesterol’s orientation with respect to the membrane normal is not considered.

### 4. RC produces native-like top-scoring models regardless of benchmark complexity

**Fig 4:**
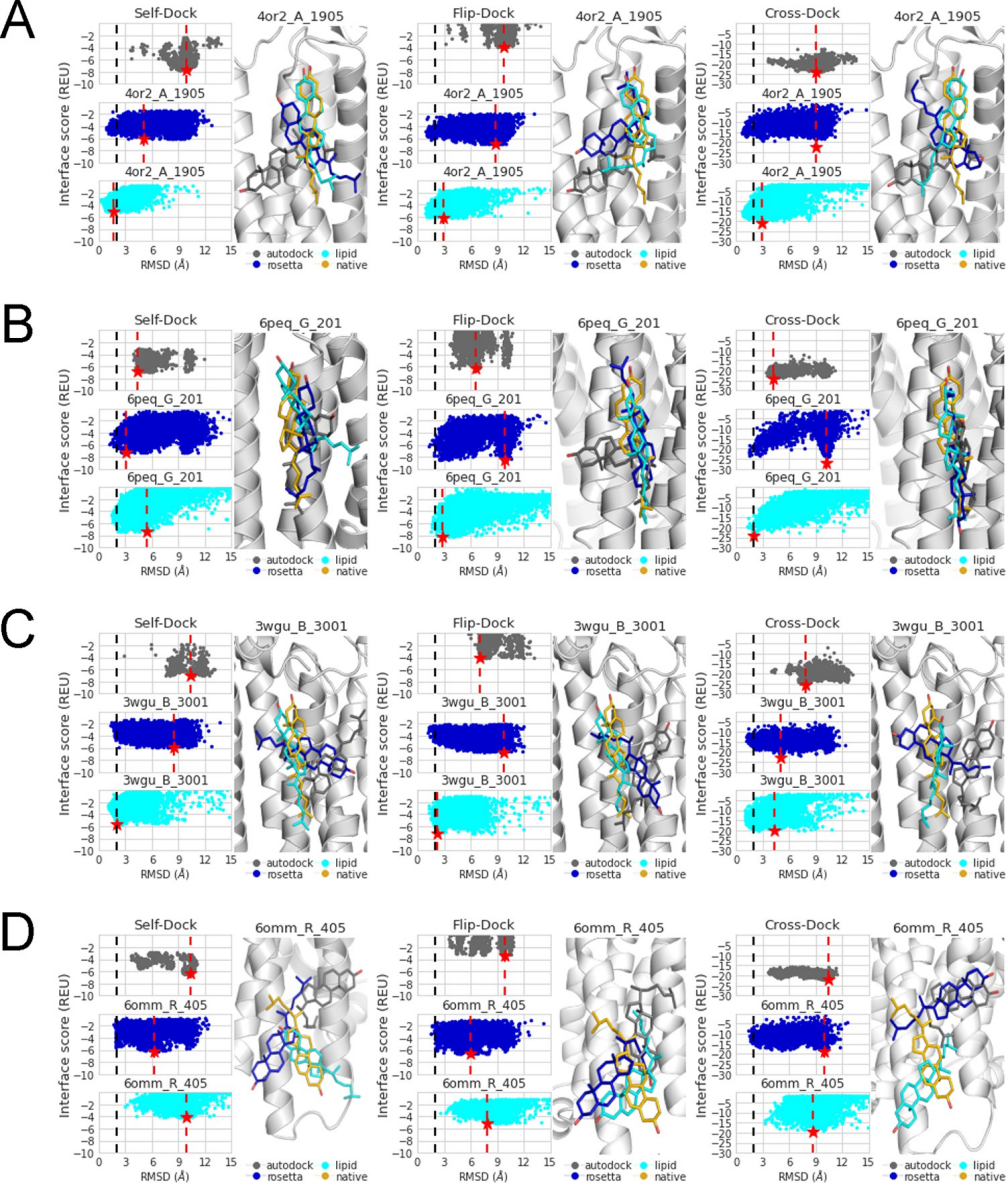
Illustrative examples of the best-scoring model in the self-dock, flip-dock, and cross-dock benchmarks. (A,B) RC (lipid) succeeds. (C,D) RC (lipid) fails. The co-crystal structure is shown (gold) aligned with the results from AutoDock (gray), RosettaLigand (blue), and RC (lipid, cyan). A black dotted line denotes the RMSD of 2 Å, and a red dotted line with a red star denotes the best-scoring model.

Although RC succeeds in modeling most of the complexes, it fails in some cases, and its ability to model certain complexes was affected by the complexity of the benchmark. In Fig 4, we show top-scoring protein-cholesterol models where RC succeeds (PDBIDs 4OR2 and 6PEQ—Fig 4A and 4B) and fails (PDBIDs 3WGU and 6OMM—Fig 4C and 4D).

In Fig 4A, RC replicates native-like models in all three benchmarks. For the PDB 4OR2 complex, the top-scoring model produced with the protocol was 2 Å better than the top-scoring model from RosettaLigand and 3 Å better than the top-scoring model from AutoDock. The RMSD difference between RC and RosettaLigand increases with benchmark complexity, highlighting an increasing improvement of RC over RosettaLigand.

In PDB 6PEQ, RC top-scoring model RMSD improved with benchmark complexity (Fig 4B), whereas the RosettaLigand top-scoring model worsened in RMSD. For the latter, two clusters were observed and became more pronounced in the cross-dock experiment. AutoDock’s top-scoring model maintains its RMSD within the 4–6 Å range, regardless of the benchmark.

For PDB 3WGU, the top-scoring model from RC worsened in RMSD with increasing benchmark complexity (Fig 4C). The RosettaLigand top model improves in RMSD.

In Fig 4D, the RosettaLigand top-scoring model had a better RMSD than both AutoDock and RC in the self-dock and flip-dock experiments (PDB 6OMM). RC never produced a native-like model (< = 2 Å), and neither did RosettaLigand nor AutoDock, irrespective of the benchmark. The models show that the cholesterol headgroup is at the membrane core or moved into the helical bundle. While two helices make up the crystallized binding site in PDB 6OMM, the accessible pocket volume is small (216 Å^2^) in comparison to the structures in our dataset (12%). The cholesterol molecule also adopts an edge-like conformation in the binding site [24]. In edge-type sites, residues only interact with cholesterol orthogonally. Residues extend around both faces and form contacts with a bias toward the β-face; the intercalation of the β-face methyl groups with branched amino acids helps to anchor cholesterol to the binding site because of the lack of helical participation. The lack of anchoring may make it difficult for the RC algorithm to dock to that area.

A comparison of all protein-cholesterol docking models is shown in Fig C in S1 Text. RosettaLigand failed in 53% of the cases due to the hydroxyl group pointing into the membrane core. Usually, when the LipidMemGrid failed to produce accurate models, the hydroxyl group pointed towards the membrane surface but was displaced in the XY plane (37%).

### 5. The RC protocol predicts the specificity of protein-cholesterol binding sites

**Fig 5:**
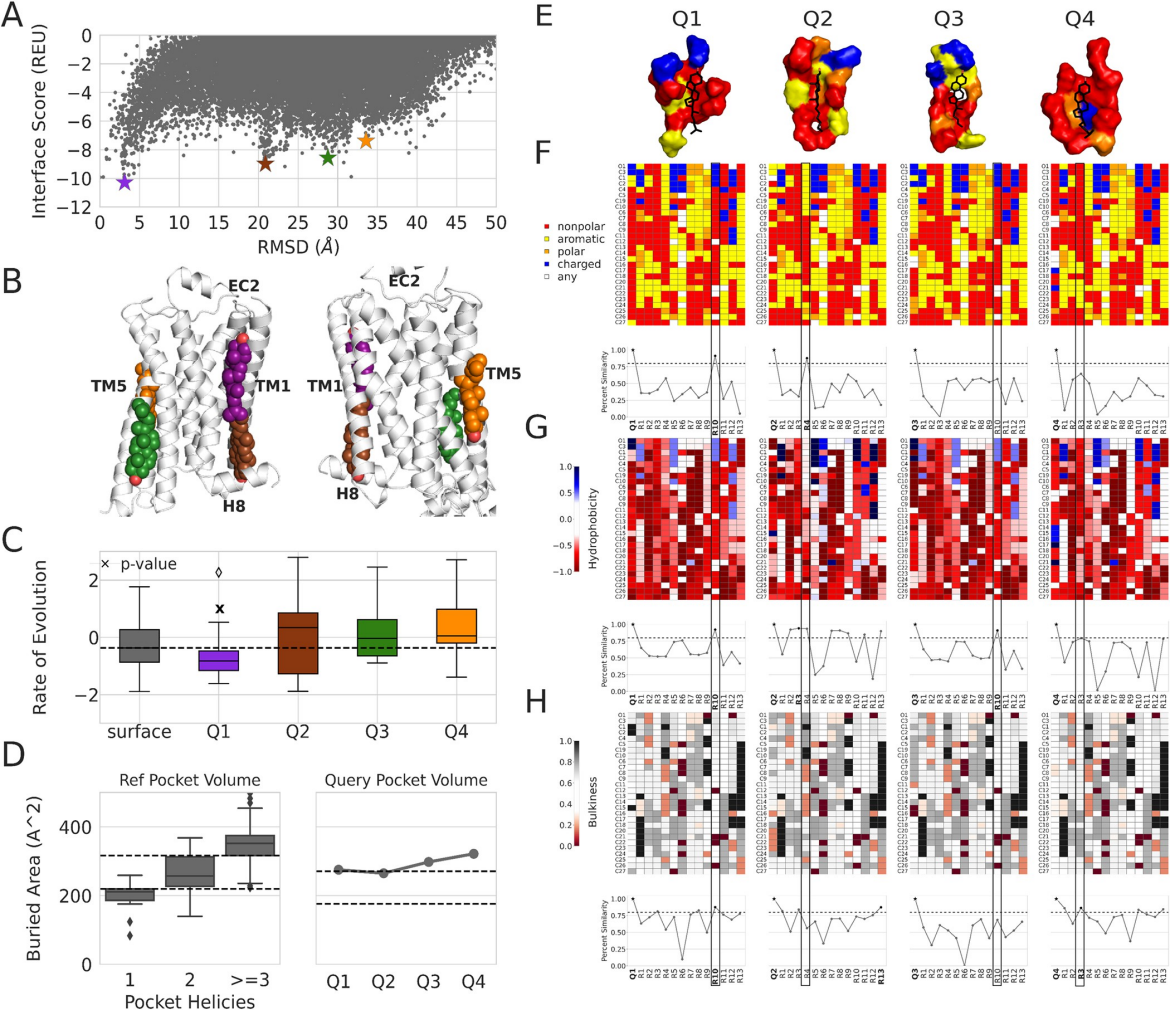
Cholesterol interaction site specificity score calculation. (A) Global docking energy landscape. (B) Cartoon representations of top query sites in each cluster. (C) Rate of evolution calculation of query binding sites. Surface residues are colored gray, and the query pockets are colored based on the stars in (A). (D) Buried Area (A^2^) of query pockets. (E) Cartoon illustration of query site pockets. (F) Residue-cholesterol 1-D fingerprints of the query pockets and percent similarity quantification. (G) Hydrophobicity of query pockets with percent similarity quantification. (H) Bulkiness of query pockets with percent similarity quantification.

Characterizing protein-cholesterol interactions is difficult due to cholesterol’s dual roles as a modulator of IMP function through direct binding (i.e. specific) and as an indirect (i.e. nonspecific) effector of membrane fluidity. Distinguishing specific binding sites from nonspecific ones remains challenging, especially if prior information is unknown. Here we use a global docking strategy with the RC protocol to predict the possible specificity of protein-cholesterol interaction sites using structural and evolutionary features. We demonstrate our approach on five proteins (see Table C in S1 Text). The results on the β2AR (PDB 2RH1) are shown in Fig 5; see Figs D-G in S1 Text for the other proteins.

First, we run global docking and identify potential binding sites on the protein via a Rosetta interface score vs. RMSD plot of the output models (Fig 5A). We visually inspected the RMSD landscape and identified funnels with multiple models clustered together. Then in each cluster, we found the lowest energy model (marked with a star) and identified that as a possible binding site. Overall, four possible binding sites were identified and analyzed. Three pockets are on the inner leaflet of the membrane, and one is on the outer leaflet side (Fig 5B).

Next, we calculate each pocket’s rate of evolution (ROE) (Fig 5C), which is compared to the baseline ROE of other lipid-accessible residues (i.e., surface) outside of the binding site. The standardized mean difference between the surface ROE and pocket ROE is taken as the Escore, where a more negative Escore indicates higher pocket conservation. Two of the pockets had ROEs less than the surface ROE, and one had a p-value of <0.1. The pocket with a significant ROE was in the outer leaflet of the membrane.

Then, we computed the pocket volume (Fig 5D). We previously determined that a cholesterol interaction site was characterized by the number of transmembrane helices involved in the interaction site and its buried area [24]. Additionally, research has shown that non-annular lipids have high-affinity interactions at grooves formed by multiple transmembrane helices and are critical for function [35–39]. All four pockets had two or more helices involved in their binding sites.

We then calculated similarity scores between the query pockets (Fig 5E) and the 13 reference pockets for physico-chemical properties of the interacting residue types (Fig 5F), hydrophobicity (Fig 5G), and bulkiness of the residues in the pockets (Fig 5H). Each 3D pocket is reduced to a 1D representation (along the cholesterol main axis) and plotted in a heatmap (Fig 5F–5H) where the cholesterol atoms (y-axis) are plotted over the pocket names (x-axis) for the query (first element on the x-axis) and reference pockets (all other elements on the x-axis). Below each heatmap is the percent similarity score where the first bold point is the query, and any other bolded points are the highest match above the cutoff of 0.8. A value of 1 is an exact match.

The best reference match with a query pocket is denoted using a black rectangle. To pick the best match across all three binding site structural characteristics (residue type physico-chemical interface, hydrophobicity, and bulkiness), we rank their matches by percent similarity. The rankings are averaged, and the highest total is chosen as the best structural match. Across the four query pockets, Q1 and Q2 matched reference pockets for all three characteristics with a percent similarity of 0.8 or above. Q3 and Q4 has a reference match above 0.8 for only one property. Then we average the binding site structural properties (pocket volume, and percent similarity of the residue interface, hydrophobicity, and bulkiness) into the Pscore.

Lastly, the Escore and Pscore are combined to create the specificity score (Table 1). The specificity score is computed as a sum of terms scaled by a weighting factor (see Methods). For the β2AR, the query specificity scores ranged from 0.54 to 0.76. The Q1 site is more likely to be specific, with a score of 0.76 over the others. Scores closest to 1 indicate possible specificity. We denote that our sample size is small, but we use a general cutoff score of 0.63 to indicate likely-specific sites. The cutoff was decided based on the average specificity score of the test set, which was 0.63. Any value above 0.63 was counted as possibly specific. The cutoff used will depend on the protein of interest.

**Table 1:** Summary of specificity score predictions.

query	Q1	Q2	Q3	Q4
color code	purple	brown	green	orange
binding site rate of evolution				
Escore^1^	-0.49	0.21	0.47	0.57
binding site interface				
physico-chemistry	0.91	0.88	0.57	0.65
hydrophobicity	0.92	0.93	0.91	0.79
bulkiness	0.87	0.56	0.69	0.86
buried area (A^2^)	1.00	0.91	0.76	0.82
Pscore^2^	0.93	0.82	0.73	0.78
specificity score^3^				
	0.76	0.62	0.54	0.55

^1^Negative scores are best

^2^Scores closest to 1 are best

^3^Possible specific sites have scores closest to 1

There is considerable evidence for both specific and nonspecific cholesterol binding sites on the β2AR [40]. Three (Q1, Q2, and Q3) of the possible binding sites have been seen in previous work. For the β2AR, cholesterol binding sites have been observed in crystal structures [41], seven have been mapped using MD simulations [42], and two have been clustered [43]. Based on Cang and Jiang’s simulations, the authors deduced biological implications for two out of the seven [42]. The sites were e1 (TM1,2,7) at the outer membrane leaflet and i2 (TM1-H8) at the inner membrane leaflet. We observed both sites from our docking experiments with a specificity score of 0.76 and 0.62, respectively. We also observed the i4 (Q3) site from Cang and Jiang. Among several class A GPCR crystal structures, such as the β2AR, a cholesterol consensus motif (CCM) was suggested between TM1-4 at the intracellular side [44]. For the CCM site, we observed docking models with an average interface score of -6.87 Rosetta energy units; however, no cluster was observed.

To summarize, our protocol enables blind cholesterol docking without prior knowledge about the binding site location. Adamian et al. separately used evolutionary selection and structural analysis to assess cholesterol binding sites where they determined that residues interacting with bound lipids are under stronger purifying selection than the other lipid facing residues [23]. We take it a step further and include energetics calculations to account for differences in binding modes of cholesterol on proteins in the RC protocol. Currently, we can only conclude that low-energy conformations of modeled cholesterol interaction sites that are evolutionarily conserved are "important" sites. For the evolutionary feature, because cholesterol is often present in the membrane space, the likelihood that a site is conserved for cholesterol is unlikely, so when it is, specificity is supposed. However, we cannot know for certain if a conserved site is specific for cholesterol. While we calculate the rate of evolution for only energetically favorable protein-cholesterol sites, other lipids could theoretically bind to those spots too. Docking other lipids to the same conserved spots as cholesterol to determine if they are as energetically favorable may be needed to improve the ranking score. In our next steps, we plan to parameterize other lipids. Ultimately our protocol should be integrated with experiments like NMR shift experiments and site-directed mutagenesis, as well as MD techniques to test our predictions of the likelihood a specific cholesterol binding site exists. Additionally, the use of cholesterol stereoisomers (*ent* and *epi*) could be utilized computationally and experimentally to conclude stereo-specificity. D’Avanzo et al. [45] and Romanenko et al. [46] showed that while *ent* and *epi* cholesterol could bind to the same site on the Kir channels, they bound in different orientations, which had differential effects on function. Overall experiments will provide useful feedback to determine the strengths and weaknesses of our method for future improvement.

## Conclusions

Here we present the RC protocol to model protein-cholesterol interactions. The RC protocol is based on RosettaLigand, where we adapt the sampling and scoring to characterize a ligand as a lipid and add an additional filtering step to predict the cholesterol binding site specificity. During the prediction phase (sampling and scoring) of the RC protocol, a different scoring grid from RosettaLigand is used during the TransformMover step. We use the LipidMemGrid, which characterizes the input ligand as a lipid, accounting for van der Waals interactions and the directionality of the lipid in the membrane. The LipidMemGrid is a general scoring grid that can be applied to any lipid as long as the head and tail atoms are specified, and a knowledge-based scoring potential is provided. Currently, the protocol has only been benchmarked on cholesterol. The filtering step of the RC protocol is specifically geared toward predicting cholesterol binding site specificity. We independently compute (1) the rate of evolution of the binding site compared to other lipid-accessible residues and (2) lipid specificity fingerprints and combine these into a specificity score.

Using the prediction phase of the RC protocol improved sampling and scoring of native poses over the RosettaLigand baseline in 91% of cases. With the complete RC protocol, we were able to recapitulate experimentally validated specific sites on the β2AR. The RC protocol is a fast and inexpensive tool that can screen a large number of possible protein-cholesterol complexes for binding sites and highlight select candidates. Our approach provides a starting point for modeling, predicting, and designing cholesterol binding sites to further examine with MD simulations and experiments.

While the LipidMemGrid is a general energy grid, an important remaining task is benchmarking it on other lipids. Lipids with various tail numbers, lengths, and saturations will change the conformational search space of the LipidMemGrid. Additional future directions include experimental validation of sites we predicted using the RC protocol and the design of specific cholesterol binding sites on proteins.

## Materials and methods

### Dataset generation

We created a dataset of 445 protein-cholesterol complexes from the OPM database. Structures determined via X-ray crystallography or Electron microscopy at a resolution smaller or equal to 3.5 Å were extracted. Only structures with cholesterol within 6 Å from their corresponding IMP were included. Several of the proteins were elucidated with multiple cholesterol molecules, and they were separated into distinct structures such that complexes in "training" and test sets were proteins with a single bound cholesterol.

We prepared the structures for docking, grid generation, and analysis in the following way: Each structure was downloaded from OPM and cleaned and renumbered. Also, missing atoms were added to incomplete residues. Next, we removed the cholesterol and repacked and minimized the protein side-chains using the RosettaFastRelaxMover. This removes any possible clashes from the starting model. Separately, to model lipid flexibility, we generate 205 cholesterol conformations using MOE [47].

Then, we randomly split the 445 complexes into a "training set" of 400 structures and a test set of 45 structures (Table A in S1 Text). We used the "training set" to generate the LipidMemGrid scoring grid (see below), and the test set to evaluate the three docking protocols: RC, RosettaLigand, and AutoDock.

### LipidMemGrid generation

The LipidMemGrid statistical energy potential was generated using 400 protein-cholesterol complexes. All 400 complexes were superimposed onto each other via their membrane coordinates, producing an average membrane representation around all the protein-cholesterol complexes. The XYZ coordinates were extracted for the O1 atom, C25 atom, and the center of mass of cholesterol. The coordinates of the O1 atom were used to orient the head group and the C25 atom to orient the tail of cholesterol. We then computed a histogram along the Z-coordinate with a length of 50 Å and a bin width of 2 Å. The average plasma membrane is ~30–40 Å thick [48]. We used a length of 50 Å to smooth the transition between the membrane and the soluble space. The propensities and energies for each bin were calculated using the inverse Boltzmann relationship [49]. The energies were normalized between -1 and 0. Cubic spline interpolation was used to fit polynomials and describe the directionality of cholesterol.

### LipidMemGrid test set

The performance of the prediction phase (TransformMover and HighResMover) in the RC protocol was evaluated in self-dock, flip-dock, and cross-dock benchmarks (Fig 1B and section 1. in Results). We compared the results from the test set of 45 protein-cholesterol complexes against AutoDock and RosettaLigand in their ability to predict the likelihood of specific cholesterol binding sites on IMPs. For each of the 45 protein-cholesterol complexes, we set up 45 docking runs with an output of 100 models, creating 202,500 models (see Table B in S1 Text). We used RosettaScripts [14,50,51] to recalculate the interface score to accurately compare the AutoDock produced models to those of RosettaLigand and RC.

We also evaluated the performance of the complete RC protocol in a global-docking benchmark using a test set of five IMPs. The IMPs in this test set included proteins with multiple experimental and MD simulation studies that predicted specific binding sites on the protein [52–61]. For each of the five proteins, cholesterol was globally docked by randomly choosing starting points from the lipid-accessible region of the protein. Six independent docking runs were set up with an output of 5,000 models, each creating 30,000 models per IMP.

### RC protocol

RosettaLigand is geared towards modeling protein-small molecule interactions. Our docking algorithm, RC, combines the prediction phase (TransformMover and HighResMover) with a novel specificity filter to model protein-cholesterol interactions. RC runs within an XML-scriptable interface [62].

In RC, the TransformMover converts the input model into a centroid representation and then uses random translation and rotation of the ligand to search the binding site using pre-computed scoring grids. Grids are generated once and are defined in the ScoringGrids block in the XML.

Our protocol combines the standard RosettaLigand energy grid (ClassicGrid) with our novel LipidMemGrid in a weighted ratio of 1:4 to find the best protein-cholesterol interfaces. Cholesterol is first translated and rotated in the binding pocket (using the TransformMover). Then, the attractive and repulsive forces in the ClassicGrid and the lipid directionality in the LipidMemGrid were computed, and translational and rotational moves were accepted in a Monte-Carlo fashion.

The LipidMemGrid calculates the orientation of the residue-lipid interface by taking a user-defined head and tail atom. For cholesterol, the head atom is O1, and the tail atom is C25. During the full-atom HighResMover stage, side-chain rotamers are refined, and the protein-lipid interface is optimized using small perturbations to the lipid in conjunction with the Metropolis Monte Carlo criteria. The protein-cholesterol complexes are scored using the *franklin2019* score function.

Lastly, we calculate the specificity likelihood score of the cholesterol interaction site, which is a weighted sum of the rate of evolution (Escore) and the structural properties of the binding site (Pscore).

### Specificity score

We define a metric to predict the possible specificity of cholesterol binding sites. This metric (specificity score) is a weighted sum of the binding site Escore (rate of evolution), which is transformed to a probability using the logistic function, and the Pscore (structural). The Escore contains the evolutionary information of the protein-cholesterol interface as compared to the lipid-accessible area on the protein that does not bind cholesterol. The Pscore represents structural information such as pocket volume, residue type physico-chemical properties, hydrophobicity, and bulkiness. The transformed Escore and the Pscore are combined into the specificity score, where the weights were empirically derived (see Protocol Capture for more detailed steps on running the RC protocol).

### Binding site rate of evolution (Escore)

The Consurf web server was used to calculate the rate of evolution, using the Bayesian method, for each amino acid in the proteins in our dataset [63–67]. A multiple sequence alignment (MSA) was calculated based on the fasta sequence of the input protein structure. MSAs were obtained by running HHMER against the UNIREF-90 sequence database [68], with minimum coverage to the query sequence set to 35%, maximum coverage set to 95%, and the E-value cutoff for inclusion set to 0.0001. We separate residues into cholesterol binding and lipid-accessible (non-cholesterol binding) categories for each protein-lipid interface using the netsurfp server [69,70] and the mp_lipid_acc method [71] in the Rosetta software suite. The Escore is computed as the standard mean difference of the ROE:

$$E_{score}=\frac{{\bar{x}}_{i}-{\bar{x}}_{j}}{\sqrt{(s_{i}^{2}+s_{j}^{2})/2}}$$

where ${\bar{x}}_{i}$ is the mean ROE of the cholesterol-binding group and ${\bar{x}}_{j}$ is the mean ROE of the lipid-accessible (non-cholesterol binding) group. And s_***i***_ is the ROE standard deviation of the cholesterol-binding group and s_***j***_ is the ROE standard deviation of the lipid-accessible (non-cholesterol binding) groups. Additionally, the Wilcoxon ranksums test was used to calculate the significance (p-value) of the Escore values.

### Binding site structure (Pscore)

We reduced the 3D geometry of the cholesterol binding sites into a 1D representation by calculating the paired distances between the protein residues and the cholesterol atoms (see Fig 5F–5H). We used the MDAnalysis [72,73] Python package to extract the protein-cholesterol binding site residues. Amino acid side-chains within a specified distance (6 Å) of the cholesterol are considered a part of the binding site. The side-chain with the closest distance to a cholesterol atom is binned as the representative residue group at that position. Residues in the binding sites are assigned one of four groups defined by the chemical properties of the possible 20 amino acids. Set-1 (aromatic: F, W, Y); Set-2 (polar-charged: D, E, K, R, H); Set-3 (non-polar: A, I, L, M, V, G); Set-4 (polar-uncharged: C, N, Q, S, T, P).

We calculated similarities for the residue type physico-chemical interface, hydrophobicity, and bulkiness between the query models and a reference set. We use an average of these four properties to calculate the overall binding site Pscore using Python3. The reference set was created by assigning each of the binding sites in our dataset residue types based on the classifications above and then grouping them using hierarchical clustering. Clusters were kept if two or more binding sites belonged to a group and two or more helices were involved in the binding site. Then we calculated the tanimoto similarity between each cluster and manually inspected each cluster to remove any redundancy. This strategy resulted in 13 clusters representing 52% of the structures in our dataset.

#### Pocket residue type physico-chemical interface similarity

We used the Jaro-Winkler distance [74,75] to compute the similarity between docked query complexes and the reference structures. A score of 1 is an exact match, and 0 is for no similarity.

$${sim}_{w}={sim}_{j}+lc(1-{sim}_{j})$$

where *sim*_*j*_ is the Jaro-Winkler similarity for 1D representations of *s*_1_ and *s*_2_; *l* is the length of common residue types at the start of the 1D representation; and *c* is a constant scaling factor for how much the score is adjusted upwards for having common residue types.

#### Pocket hydrophobicity similarity

The hydrophobicity index is a metric for how soluble an amino acid is in water. Hydrophobic residues are expected to be found in the interior of an IMP or the lipid-exposed surface in a membrane environment. The hydrophobicity of each residue in the binding site was determined using the unified hydrophobicity scale [76]. The cosine similarity was calculated to determine pocket hydrophobicity similarity.

$$cos(i,j)=\frac{i*j}{\left|\left|i\right||*|\left|j\right|\right|}$$

where 1−*cos*(*i*, *j*) is the percent similarity for hydrophobicity of *i*, the query pocket, and *j*, each of the reference pockets.

#### Pocket bulkiness similarity

Cholesterol contains four rings which can make CH-π interactions with aromatic residues and anchor cholesterol to the protein. The molecule also contains two methyl groups helping to intercalate with branched residues for anchoring. Since aromatic and branched residues are some of the bulkiest, we account for their possible interactions by calculating their molecular volume (A^3) in the binding site. The molecular volume (A^3) of each amino acid was determined using Zamyatnin et al. [77] definitions. We used the cosine similarity to compute the bulkiness similarity between the query complexes and the reference set.

#### Pocket volume

Cholesterol tends to interact with the surface of the protein in transient and lasting ways. We expect that the deeper the surface groove, the more likely a cholesterol molecule will bind to the protein and make a lasting interaction. In previous work, we showed that the buried surface area (BSA) and the number of transmembrane helices involved in the binding pocket correlate with a likely-specific interaction site [24]. The BSA of each complex was calculated using the Shrake–Rupley algorithm-based tool (dr_sasa) [78].

### AutoDock Protocol

AutoDock is one of the most cited molecular docking programs [15]–it was used as a baseline to evaluate the performance of RC in our three benchmarks (self-dock, flip-dock, and cross-dock). The test set proteins and corresponding cholesterol molecules were reformatted and cleaned according to the current RosettaLigand protocol before use with AutoDock. We used AutoDockTools to convert the PDBs into their corresponding PDBQT format. The Grid box size used was 60 Å × 60 Å × 60 Å. Weighting factors appropriate for a membrane environment were used according to Lee et al. [79]. All other parameters were kept to their default settings. Docking was performed using the Lamarckian Genetic algorithm with 100 separate runs (*ga_run* = 100) a population size of 350 (*ga_pop_size* = 350). The models were re-scored using RosettaScripts to compare the scores to those from the RC and the RosettaLigand protocols.

## Supporting information

S1 Text **Table A.** RC benchmark dataset. **Table B.** Docking benchmark setups. **Table C.** Global docking dataset. **Fig A.** Protein-cholesterol RMSD distribution for the (A-B) self-dock, (C-D) flip-dock, and (E-F) cross-dock benchmarks. RMSD values range from 0 to 18 Å. AutoDock models are gray, RosettaLigand models are blue, and RC (lipid) models are cyan. The red dotted lines mark 2 Å. **Fig B.** β2-adrenergic receptor sampled conformations using AutoDock. The native cholesterol conformation is shown in gray (-2.34 Rosetta interface score, -7.09 AutoDock free energy of binding). Other (black) sampled cholesterol conformations in a horizontal (-2.62 Rosetta interface score, -7.05 AutoDock free energy of binding) or flipped (3.51 Rosetta interface score, -7.22 AutoDock free energy of binding) conformation. Residues making polar contacts are colored green. **Fig C.** Individual protein-cholesterol interface score versus RMSD plots for the (A) self-dock, (B) flip-dock, and (C) cross-dock benchmarks. RMSD values range from 0 to 18 Å. AutoDock models are gray, RosettaLigand models are blue, and RC (lipid) models are cyan. A black dotted line denotes the 2 Å RMSD, and a red dotted line with a red star denotes the lowest scoring model. **Fig D.** NaK ATPase cholesterol interaction site specificity score. (A) Global docking energy landscape. (B) Cartoon representations of top query sites in each cluster. (I) Table of specificity scores. **Fig E.** μ-Opioid receptor cholesterol interaction site specificity score. (A) Global docking energy landscape. (B) Cartoon representations of top query sites in each cluster. (I) Table of specificity scores. **Fig F.** Serotonin 2B receptor cholesterol interaction site specificity score. (A) Global docking energy landscape. (B) Cartoon representations of top query sites in each cluster. (I) Table of specificity scores. **Fig G.** γ-Secretase cholesterol interaction site specificity score. (A) Global docking energy landscape. (B) Cartoon representations of top query sites in each cluster. (I) Table of specificity scores.
(DOCX)
