# Supplementary material for: Docking cholesterol to integral membrane proteins with Rosetta
Source: PLoS Comput Biol. 2023 Mar 27;19(3):e1010947. doi: 10.1371/journal.pcbi.1010947 (PMC10042369; doi:10.1371/journal.pcbi.1010947)
Supplement: S1 Text — Table A. RC benchmark dataset. Table B. Docking benchmark setups. Table C. Global docking dataset. Fig A. Protein-cholesterol RMSD distribution for the (A-B) self-dock, (C-D) flip-dock, and (E-F) cross-dock benchmarks. RMSD values range from 0 to 18 Å. AutoDock models are gray, RosettaLigand models are blue, and RC (lipid) models are cyan. The red dotted lines mark 2 Å. Fig B. β2-adrenergic receptor sampled conformations using AutoDock. The native cholesterol conformation is shown in gray (-2.34 Rosetta interface score, -7.09 AutoDock free energy of binding). Other (black) sampled cholesterol conformations in a horizontal (-2.62 Rosetta interface score, -7.05 AutoDock free energy of binding) or flipped (3.51 Rosetta interface score, -7.22 AutoDock free energy of binding) conformation. Residues making polar contacts are colored green. Fig C. Individual protein-cholesterol interface score versus RMSD plots for the (A) self-dock, (B) flip-dock, and (C) cross-dock benchmarks. RMSD values range from 0 to 18 Å. AutoDock models are gray, RosettaLigand models are blue, and RC (lipid) models are cyan. A black dotted line denotes the 2 Å RMSD, and a red dotted line with a red star denotes the lowest scoring model. Fig D. NaK ATPase cholesterol interaction site specificity score. (A) Global docking energy landscape. (B) Cartoon representations of top query sites in each cluster. (I) Table of specificity scores. Fig E. μ-Opioid receptor cholesterol interaction site specificity score. (A) Global docking energy landscape. (B) Cartoon representations of top query sites in each cluster. (I) Table of specificity scores. Fig F. Serotonin 2B receptor cholesterol interaction site specificity score. (A) Global docking energy landscape. (B) Cartoon representations of top query sites in each cluster. (I) Table of specificity scores. Fig G. γ-Secretase cholesterol interaction site specificity score. (A) Global docking energy landscape. (B) Cartoon representations of top query sites in each [file pcbi.1010947.s001.docx]

S1 Text

**Docking Cholesterol to Integral Membrane Proteins with Rosetta**

Brennica Marlow^1,2^, Georg Kuenze^1,3,4^, Jens Meiler^1,2,3,4*^ and Julia Koehler Leman^5^*

^1^Center for Structural Biology, Vanderbilt University, Nashville, Tennessee, United States of America; ^2^Chemical and Physical Biology Program, Vanderbilt University, Nashville, Tennessee, United States of America; ^3^Department of Chemistry, Vanderbilt University, Nashville, Tennessee, United States of America; ^4^Institute for Drug Discovery, Leipzig University Medical School, Leipzig, Germany; ^5^Center for Computational Biology, Flatiron Institute, Simons Foundation, New York, New York

*corresponding authors: jens@meilerlab.org, julia.koehler.leman@gmail.com

Table A. RC benchmark dataset

| Protein-Cholesterol complex | Membrane leaflet | Membrane Thickness (Å) | Protein Family |
| --- | --- | --- | --- |
| 2rh1_C_413 | C | 31.8 | GPCR class A |
| 2zxe_A_3001 | C | 31.9 | P-ATPase |
| 3d4s_A_403 | C | 33.8 | GPCR class A |
| 3ny9_A_1201 | C | 33.8 | GPCR class A |
| 3wgu_B_3001 | C | 30.8 | P-ATPase |
| 4eiy_A_2405 | E | 32.2 | GPCR class A |
| 4ntj_A_1202 | E | 31.8 | GPCR class A |
| 4or2_A_1902 | E | 31.4 | GPCR Class C |
| 4or2_A_1905 | E | 31.4 | GPCR Class C |
| 4res_A_2002 | E | 30 | P-ATPase |
| 4xt1_A_401 | E | 31.6 | GPCR class A |
| 5avz_B_3001 | C | 31.9 | P-ATPase |
| 5aw7_B_3001 | C | 31.9 | P-ATPase |
| 5aw8_B_3001 | C | 31.9 | P-ATPase |
| 5d5a_A_1208 | C | 31.4 | GPCR class A |
| 5k2a_A_1205 | E | 32.2 | GPCR class A |
| 5k2b_A_1205 | E | 32.2 | GPCR class A |
| 5olz_A_1205 | E | 32.2 | GPCR class A |
| 5om1_A_1204 | E | 32.2 | GPCR class A |
| 6a94_B_3003 | C | 34.2 | GPCR class A |
| 6b73_A_2002 | E | 33 | GPCR class A |
| 6co7_A_3000 | E | 32.2 | Transient Receptor Potential channel |
| 6iyc_C_303 | EI | 31 | Gamma Secretase |
| 6lpj_A_2405 | E | 32.2 | GPCR class A |
| 6lpk_A_2405 | E | 32.2 | GPCR class A |
| 6lpl_A_2404 | E | 32.2 | GPCR class A |
| 6omm_R_405 | E | 30.6 | GPCR class A |
| 6pb1_P_401 | E | 32.2 | GPCR Secretin B |
| 6pb1_P_404 | C | 32.2 | GPCR Secretin B |
| 6peq_G_201 | E | 30.6 | Glutamate-gated Ion Channel (GIC) family |
| 6ps6_A_1206 | C | 33.8 | GPCR class A |
| 6pt2_A_1201 | E | 34.2 | GPCR class A |
| 6s0q_A_1214 | E | 32.2 | GPCR class A |
| 6s7p_A_1307 | C | 35 | Multidrug Resistence Exporter (MDR) |
| 6s7p_A_1309 | C | 35 | Multidrug Resistence Exporter (MDR) |
| 6uy0_A_1606 | C | 32.2 | Drug Conjugate Transprter (ABC C family) |
| 6v22_A_1112 | E | 32.2 | Slo Potassium Channels |
| 6wbg_B_405 | E | 37 | Pannexin |
| 6wbn_G_404 | E | 37 | Pannexin |
| 6wbn_G_405 | E | 37 | Pannexin |
| 6wiv_A_908 | C | 31.4 | GPCR Metabotropic Receptor C |
| 6zdr_A_1203 | E | 32.2 | GPCR class A |
| 6zdr_A_1205 | E | 32.2 | GPCR class A |
| 6zdv_A_1204 | E | 32.2 | GPCR class A |
| 7cfm_R_402 | C | 31.4 | GPCR class A |

Table B. Docking benchmark setups

| Benchmark | Input Structures | Model outputs | Number of runs | Total data points |
| --- | --- | --- | --- | --- |
| self-dock | 45 | 100 | 45 | 202,500 |
| flip-dock | 45 | 100 | 45 | 202,500 |
| cross-dock | 2025 | 100 | 1 | 202,500 |
| global-dock | 5 | 5000 | 6 | 150,000 |

Table C. Global docking dataset

| PDB | Membrane  Thickness (Å) | Protein Family |
| --- | --- | --- |
| 2rh1 | 31.8 | GPCR class A |
| 2zxe | 31.9 | P-ATPase |
| 4ib4 | 34 | GPCR class A |
| 5c1m | 34.4 | GPCR class A |
| 6iyc | 31 | Gamma Secretase |


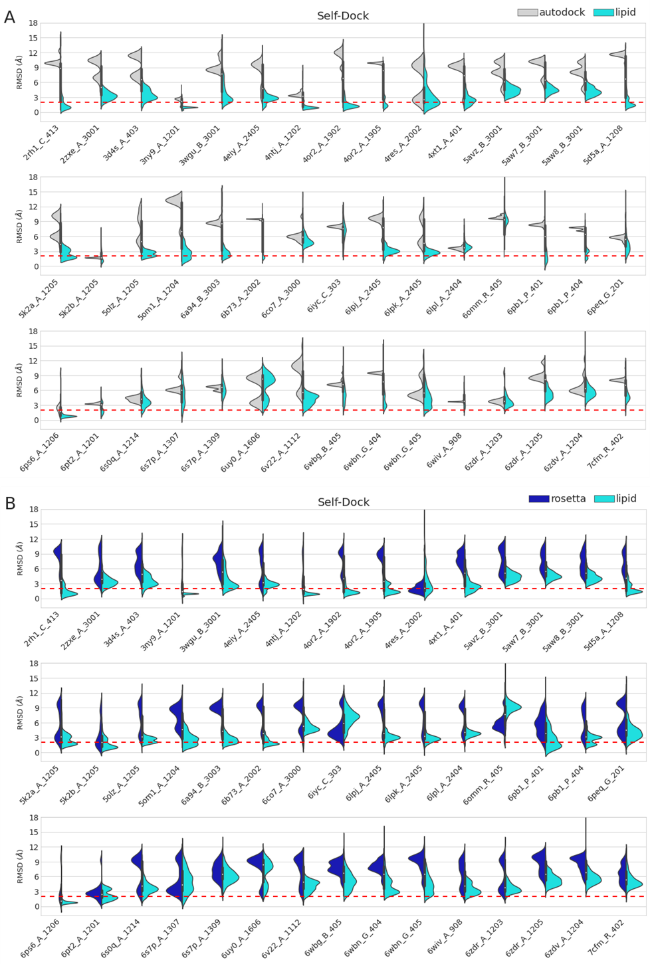


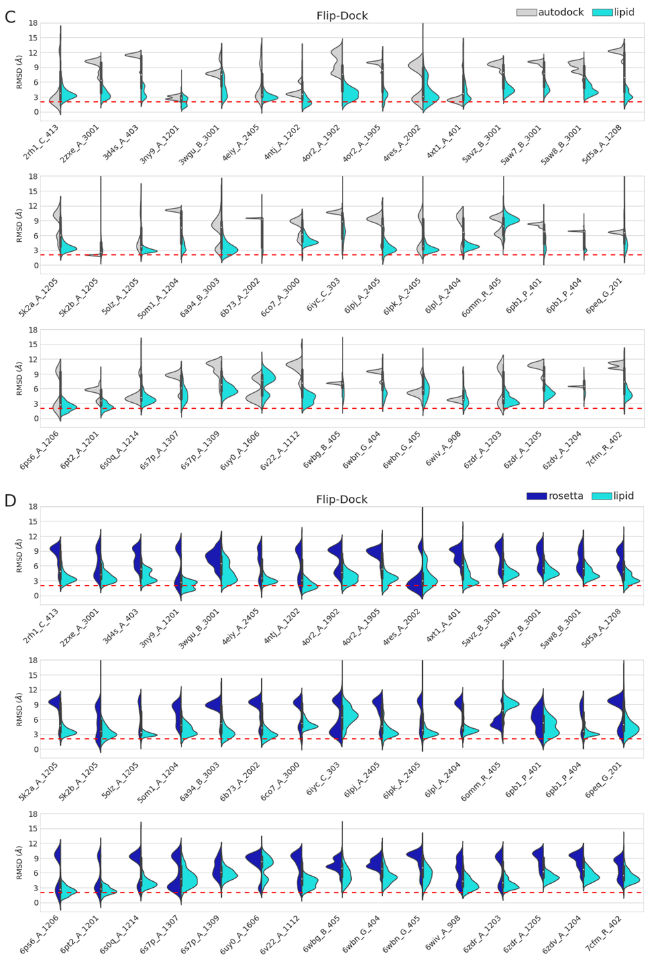


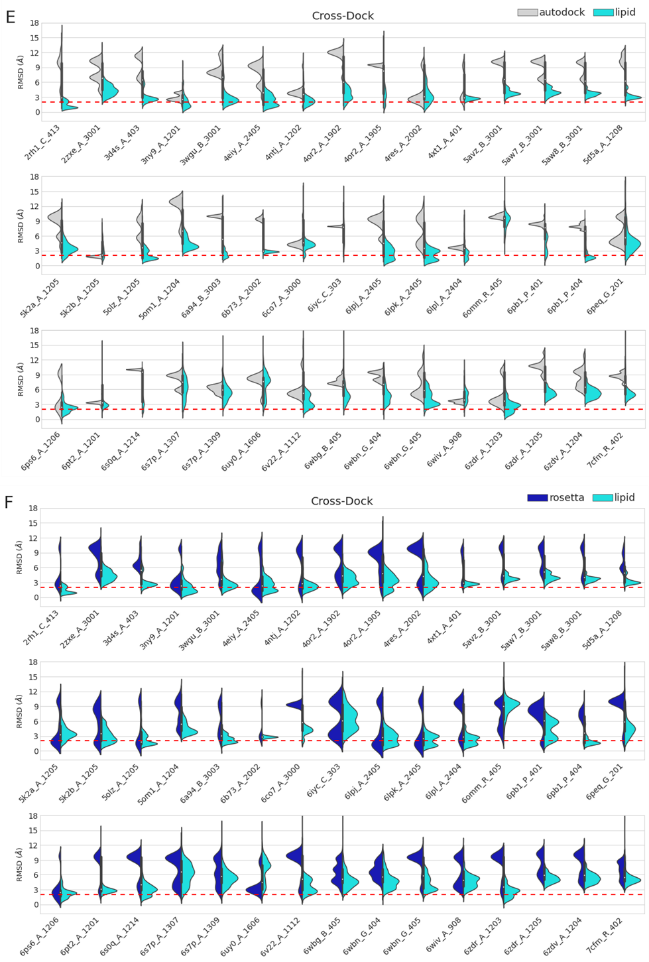


Fig A. Protein-cholesterol RMSD distribution for the (A-B) self-dock, (C-D) flip-dock, and (E-F) cross-dock benchmarks. RMSD values range from 0 to 18 Å. AutoDock models are gray, RosettaLigand models are blue, and RC (lipid) models are cyan. The red dotted lines mark 2 Å.

RC is inherently biased towards recapitulating vertical (parallel to the membrane normal) cholesterol binding conformations because it was “trained” on mostly vertical cholesterol conformations. With the implicit Rosetta membrane and the LipidMemGrid scoring grid it would be difficult for it to sample horizontal (perpendicular to the membrane normal) conformations often. For AutoDock we used the weighting factors for hydrogen bonds and hydrophobic effects to try and simulate a membrane environment as shown in Lee et al. (75). However, lack of bias towards the vertical conformations and the lack of a membrane environment allows AutoDock to sample the horizontal conformation more often. AutoDock in general samples more conformations (funnels) away and near the putative native structure as shown in Figure 2. Additionally, seeing as cholesterol has a small head group region AutoDock rescues hydrogen bonding with residues at the membrane core.


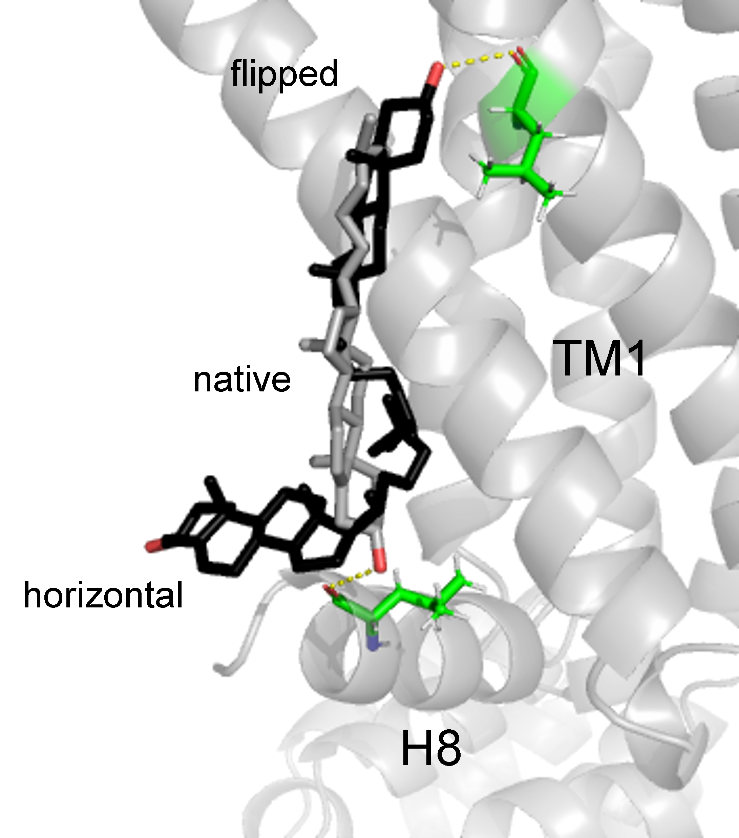


Fig B. β2-adrenergic receptor sampled conformations using AutoDock. The native cholesterol conformation is shown in gray (-2.34 Rosetta interface score, -7.09 AutoDock free energy of binding). Other (black) sampled cholesterol conformations in a horizontal (-2.62 Rosetta interface score, -7.05 AutoDock free energy of binding) or flipped (3.51 Rosetta interface score, -7.22 AutoDock free energy of binding) conformation. Residues making polar contacts are colored green.


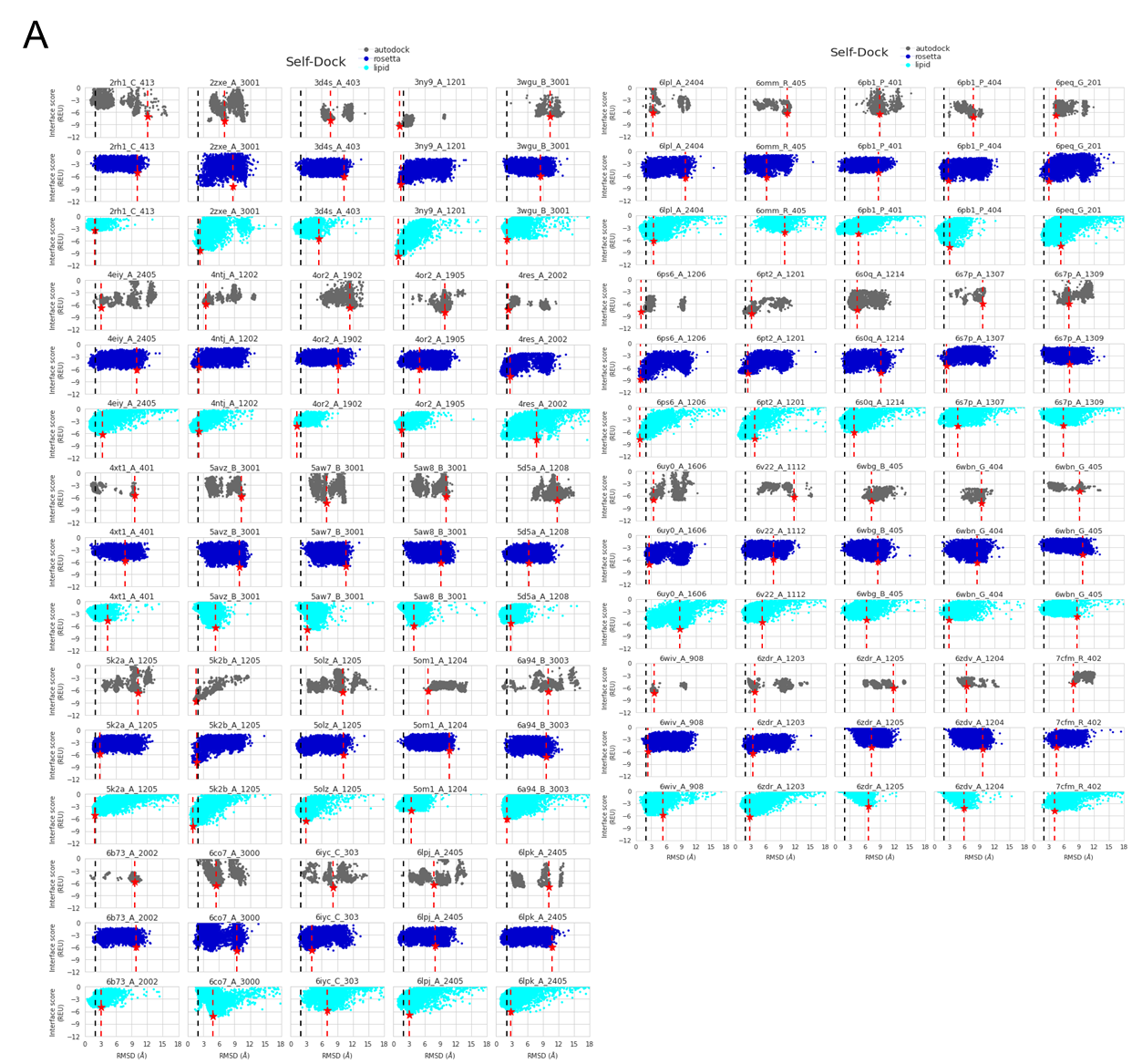

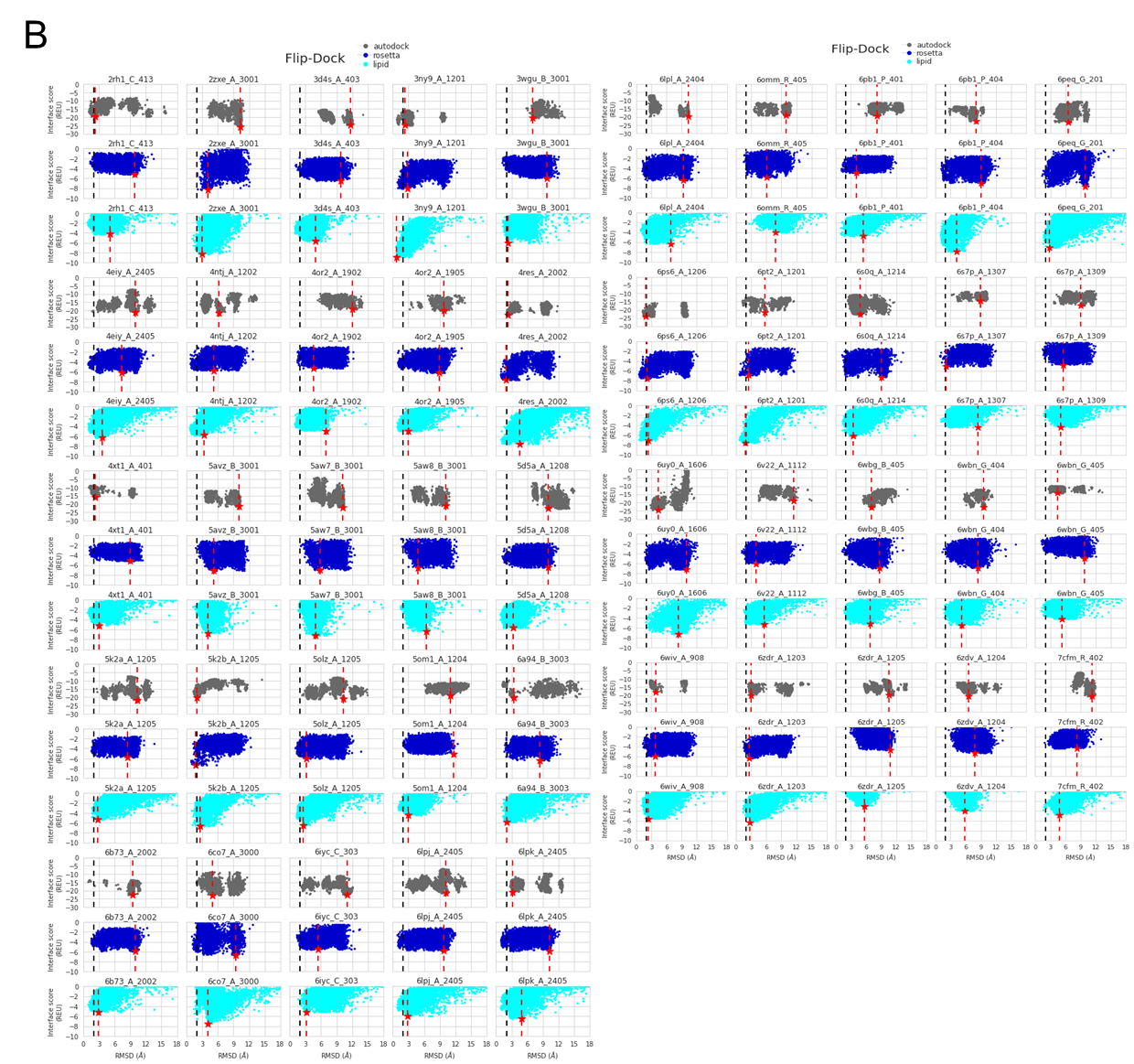


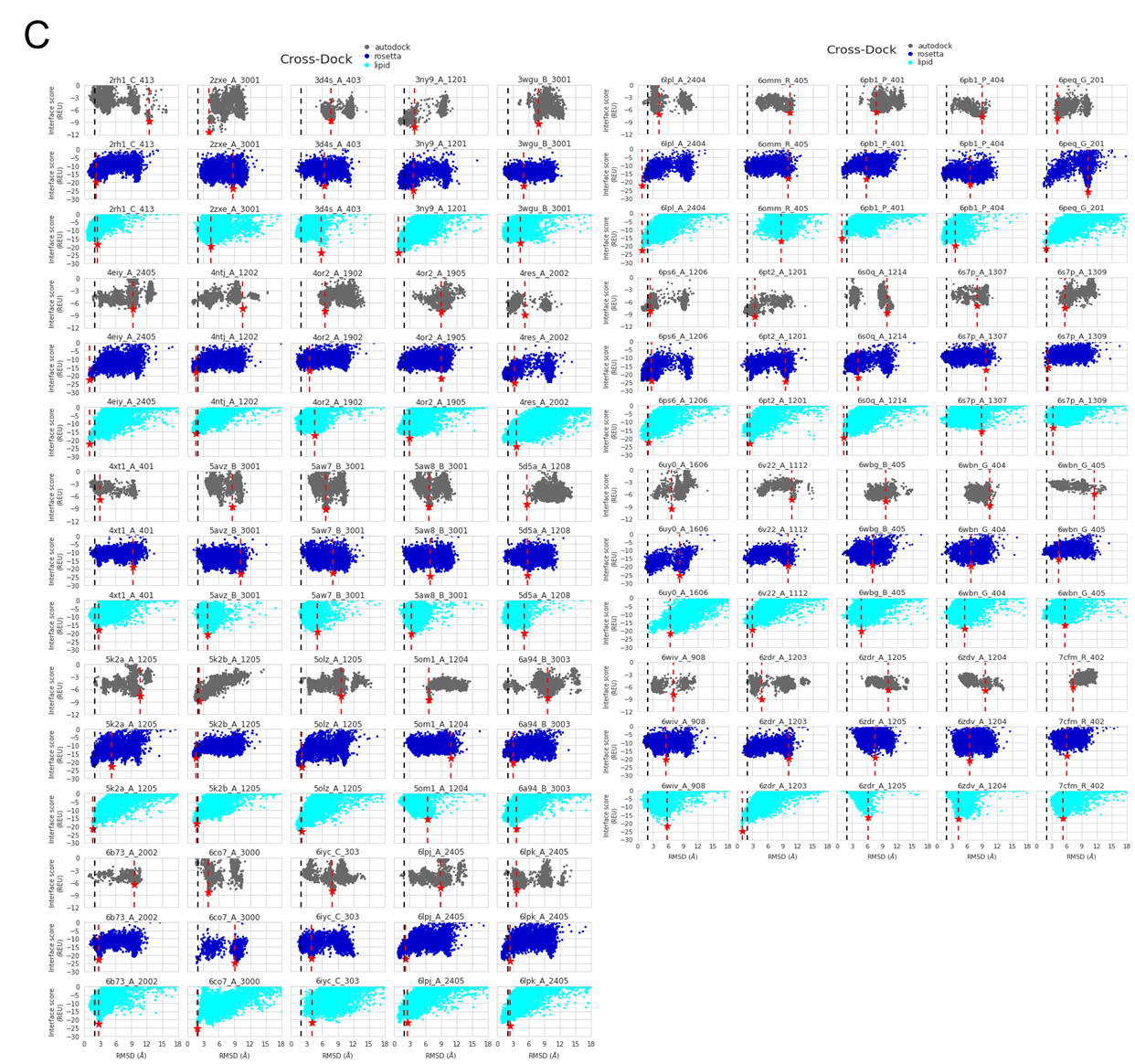


Fig C. Individual protein-cholesterol interface score versus RMSD plots for the (A) self-dock, (B) flip-dock, and (C) cross-dock benchmarks. RMSD values range from 0 to 18 Å. AutoDock models are gray, RosettaLigand models are blue, and RC (lipid) models are cyan. A black dotted line denotes the 2 Å RMSD, and a red dotted line with a red star denotes the lowest scoring model.


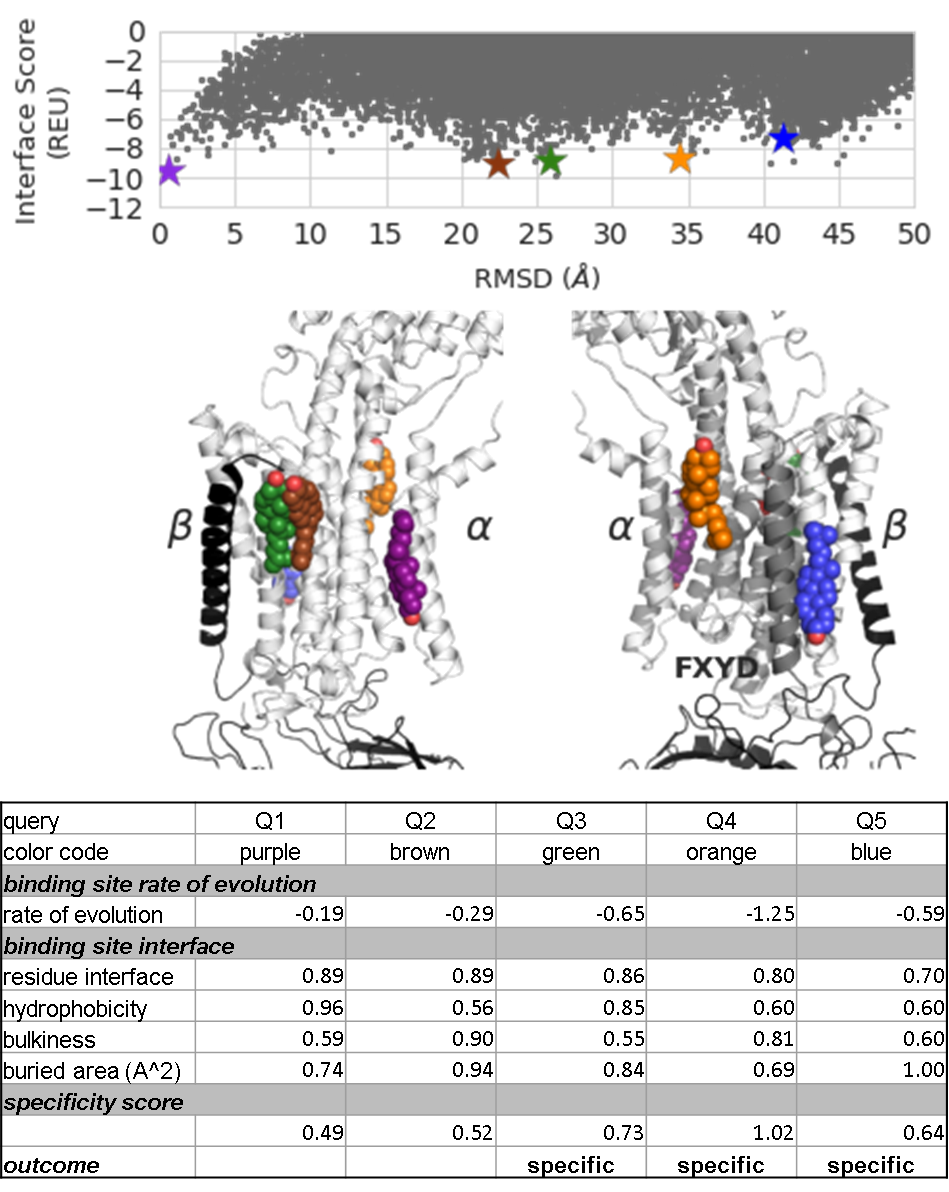


| query | Q1 | Q2 | Q3 | Q4 | Q5 |
| --- | --- | --- | --- | --- | --- |
| color code | purple | brown | green | orange | blue |
| ***binding site rate of evolution*** | | |  |  |  |
| Escore | 0.04 | -0.07 | -0.43 | -1.02 | -0.34 |
| ***binding site interface*** | |  |  |  |  |
| physico-chemistry | 0.89 | 0.89 | 0.86 | 0.80 | 0.69 |
| hydrophobicity | 0.97 | 0.59 | 0.87 | 0.63 | 0.65 |
| bulkiness | 0.59 | 0.90 | 0.55 | 0.81 | 0.64 |
| buried area (A^2^) | 0.74 | 0.94 | 0.84 | 0.69 | 1.00 |
| Pscore | 0.80 | 0.83 | 0.78 | 0.73 | 0.75 |
| ***specificity score*** | |  |  |  |  |
|  | 0.63 | 0.66 | 0.68 | 0.73 | 0.66 |

Fig D. NaK ATPase cholesterol interaction site specificity score. (A) Global docking energy landscape. (B) Cartoon representations of top query sites in each cluster. (I) Table of specificity scores.


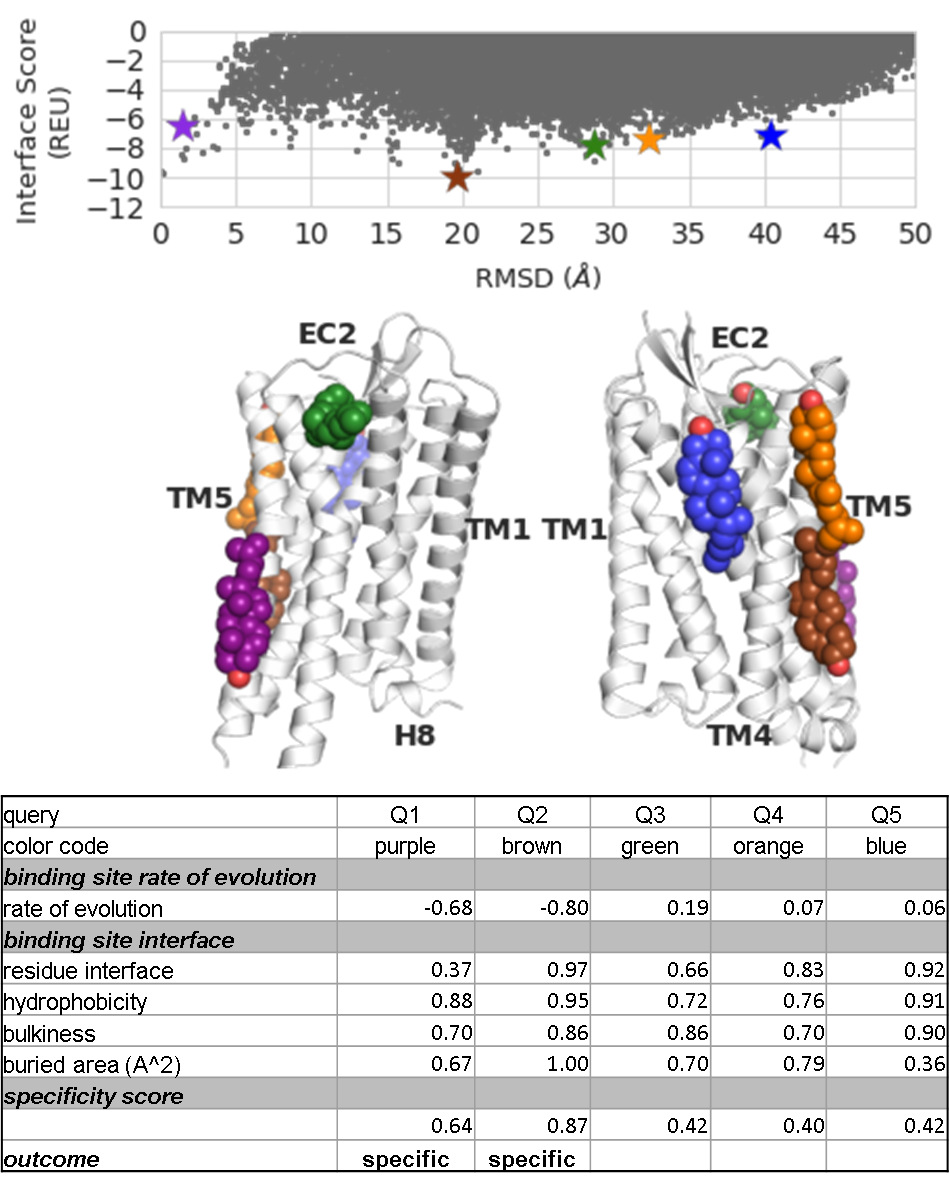


| query | Q1 | Q2 | Q3 | Q4 | Q5 |
| --- | --- | --- | --- | --- | --- |
| color code | purple | brown | green | orange | blue |
| ***binding site rate of evolution*** |  |  |  |  |  |
| Escore | -0.67 | -1.04 | 0.22 | -0.21 | -0.51 |
| ***binding site interface*** |  |  |  |  |  |
| physico-chemistry | 0.44 | 0.97 | 0.68 | 0.84 | 0.92 |
| hydrophobicity | 0.59 | 0.95 | 0.72 | 0.76 | 0.91 |
| bulkiness | 0.53 | 0.86 | 0.86 | 0.70 | 0.90 |
| buried area (A^2^) | 0.67 | 1.00 | 0.70 | 0.79 | 0.36 |
| Pscore | 0.55 | 0.95 | 0.74 | 0.77 | 0.77 |
| ***specificity score*** |  |  |  |  |  |
|  | 0.61 | 0.83 | 0.58 | 0.65 | 0.69 |

Fig E. μ-Opioid receptor cholesterol interaction site specificity score. (A) Global docking energy landscape. (B) Cartoon representations of top query sites in each cluster. (I) Table of specificity scores.


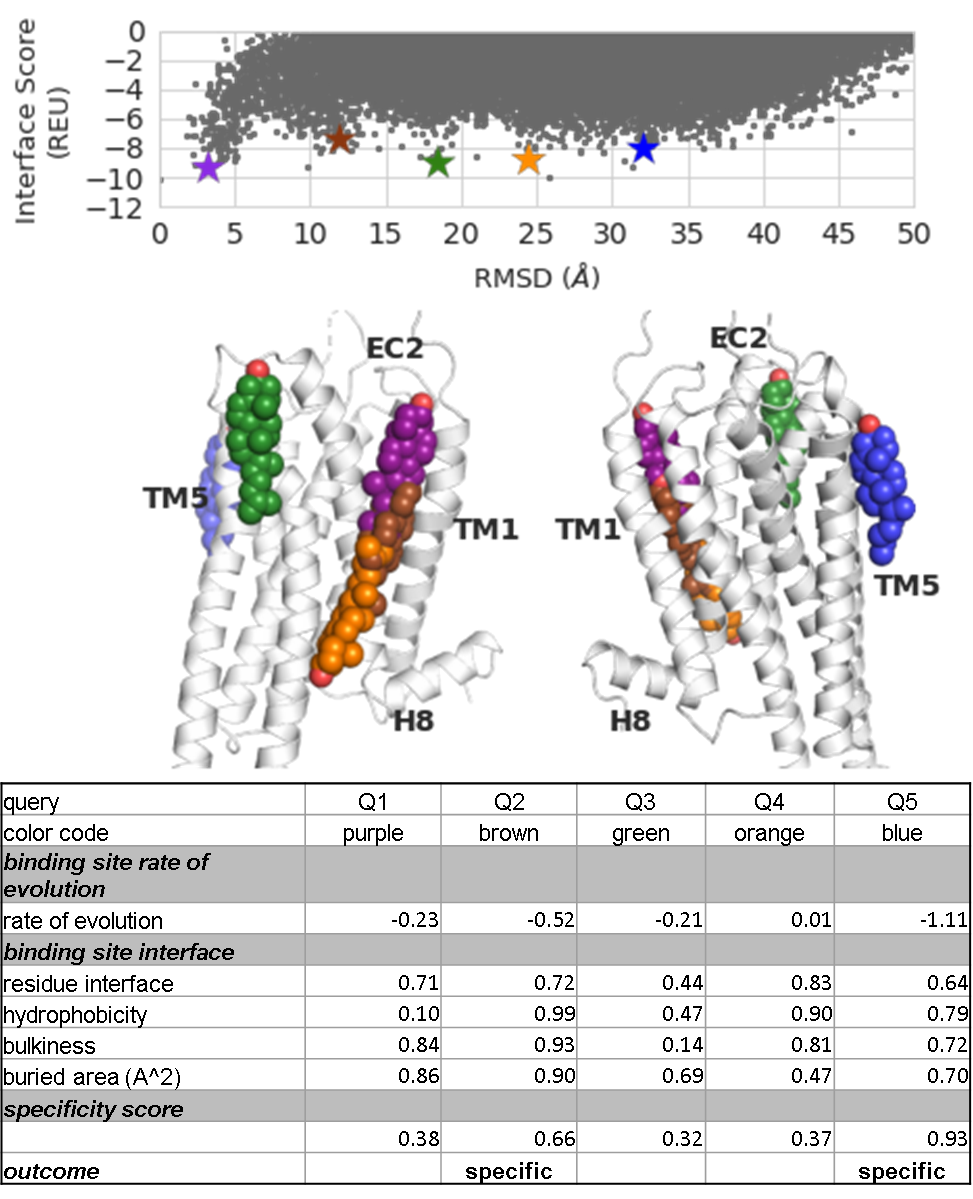


| query | Q1 | Q2 | Q3 | Q4 | Q5 |
| --- | --- | --- | --- | --- | --- |
| color code | purple | brown | green | orange | blue |
| ***binding site rate of evolution*** |  |  |  |  |  |
| Escore | -0.24 | 0.37 | 0.85 | 0.89 | -0.12 |
| ***binding site interface*** |  |  |  |  |  |
| physico-chemistry | 0.71 | 0.72 | 0.68 | 0.83 | 0.64 |
| hydrophobicity | 0.10 | 0.99 | 0.40 | 0.90 | 0.79 |
| bulkiness | 0.84 | 0.93 | 0.75 | 0.81 | 0.72 |
| buried area (A^2^) | 0.86 | 0.90 | 0.69 | 0.47 | 0.70 |
| Pscore | 0.63 | 0.88 | 0.63 | 0.75 | 0.71 |
| ***specificity score*** |  |  |  |  |  |
|  | 0.59 | 0.62 | 0.45 | 0.50 | 0.61 |

Fig F. Serotonin 2B receptor cholesterol interaction site specificity score. (A) Global docking energy landscape. (B) Cartoon representations of top query sites in each cluster. (I) Table of specificity scores.


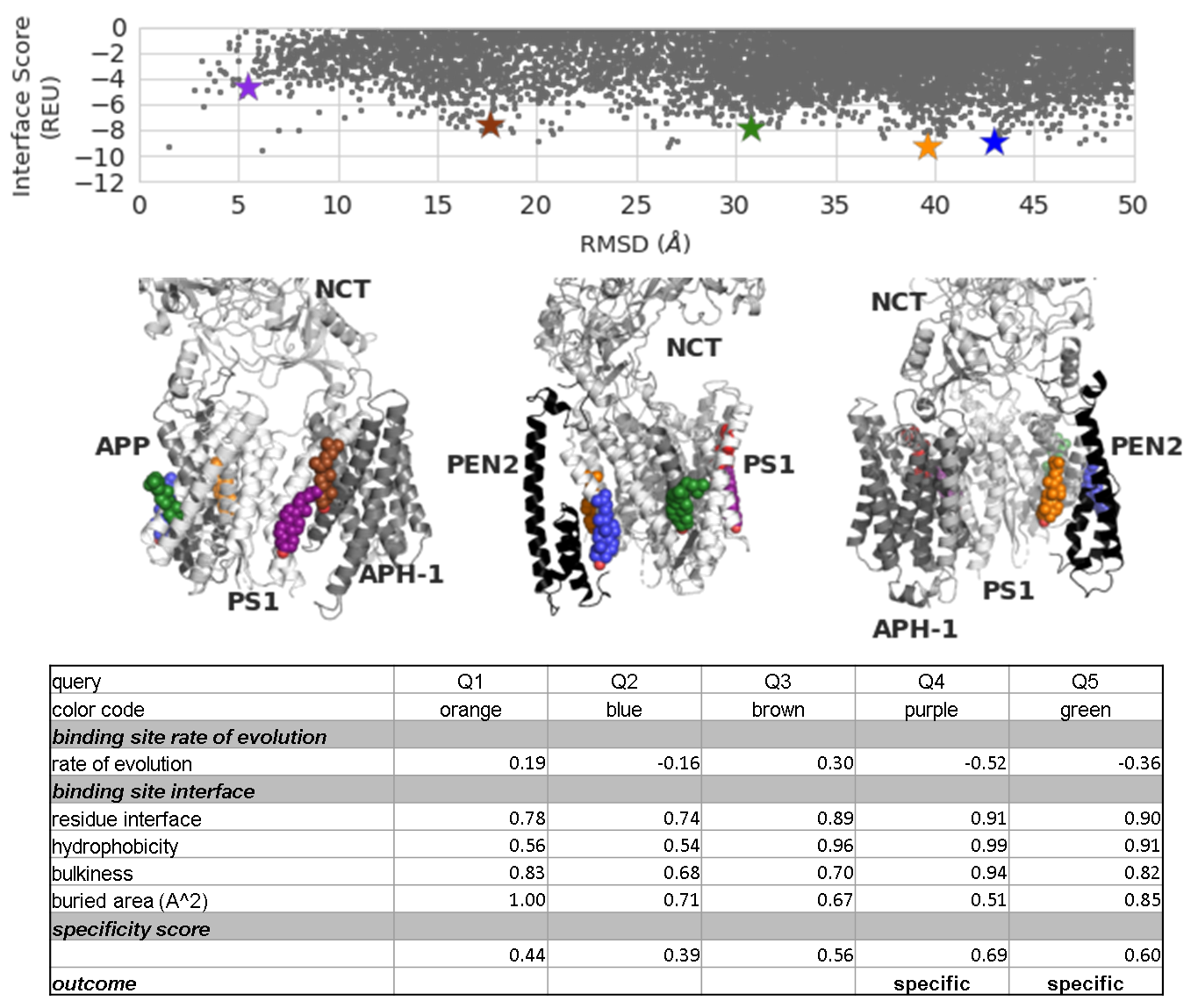


| query | Q1 | Q2 | Q3 | Q4 | Q5 |
| --- | --- | --- | --- | --- | --- |
| color code | purple | brown | green | orange | blue |
| ***binding site rate of evolution*** |  |  |  |  |  |
| Escore | -0.74 | 0.06 | -0.21 | 0.05 | -0.42 |
| ***binding site interface*** |  |  |  |  |  |
| physico-chemistry | 0.91 | 0.89 | 0.70 | 0.66 | 0.74 |
| hydrophobicity | 0.99 | 0.96 | 0.94 | 0.74 | 0.54 |
| bulkiness | 0.94 | 0.70 | 0.80 | 0.38 | 0.68 |
| buried area (A^2^) | 0.51 | 0.67 | 0.85 | 1.00 | 0.71 |
| Pscore | 0.84 | 0.81 | 0.83 | 0.70 | 0.67 |
| ***specificity score*** |  |  |  |  |  |
|  | 0.75 | 0.63 | 0.68 | 0.58 | 0.63 |

Fig G. γ-Secretase cholesterol interaction site specificity score. (A) Global docking energy landscape. (B) Cartoon representations of top query sites in each cluster. (I) Table of specificity scores.

RosettaCholesterol Protocol Capture

***Prediction (TransformMover and HighResMover)***

This protocol capture describes the RosettaCholesterol protocol. Rosetta can be obtained through www.rosettacommons.org. The following protocol has been tested with Rosetta version ad2739a. Here the example structure is the Serotonin 2B receptor crystallized in a complex with cholesterol (PDB: 4IB4). The complex was downloaded from https://opm.phar.umich.edu. All files are included in the Rosetta/main/demos/protocol_capture/rosetta_cholesterol

**Input/**

The following files are included in the **inputs/** directory to run the Prediction protocol:

| 4ib4.pdb | Serotonin 2B PDB downloaded from OPM |
| --- | --- |
| 4ib4_A.pdb | Cleaned protein |
| 4ib4_A_relax.pdb | Relaxed protein |
| 4ib4_A_dock.pdb | Combined protein and cholesterol for docking input |
| 4ib4_A_positions | Lipid accessible regions for global docking  (https://rosie.graylab.jhu.edu/mp_lipid_acc) |
| 4ib4_A.span^1^ | Rosetta spanfile |
| CLR.params^2^ | Rosetta params file for cholesterol |
| CLR.pdb | Cholesterol pdb file |
| CLR_conformers.pdb^3^ | Cholesterol conformer library |
| CLR_conformers.sdf | Cholesterol conformer output from MOE |
| dock.sh | Rosetta run command for docking |
| dock.xml | RosettaScripts XML file for docking |
| relax.sh | Rosttea run command for relaxing the protein |
| relax_wo_clr.xml | RosettaScripts XML file for relaxing the protein |

^1^Indicates protein segments that should be embedded in the membrane.

^2^Pre-computed information about the geometry and chemical features of the cholesterol.

^3^Necessary for ligand flexibility.

**Model Preparation**

Before docking in Rosetta, we must prepare the protein and cholesterol separately.

*Protein*

#1 The PDB file is cleaned and renumbered for use in Rosetta.

*Rosetta/tools/protein_tools/scripts/clean_pdb.py 4ib4.pdb A*

**Output: 4ib4_A.pdb**

#2 Generate a spanfile from the cleaned PDB to indicate the transmembrane spanning regions.

*Rosetta/main/source/bin/span_from_pdb.linuxgccrease -in:file:s 4ib4_A.pdb*

**Output: 4ib4_A.span**

#3 Refine the protein to correct local errors and optimize the membrane position. The protein is relaxed without cholesterol.

## relax_wo_clr.sh command

Rosetta/main/source/bin/rosetta_scripts.linuxgccrelease

-in:file:s 4ib4_A.pdb

-mp:setup:spanfiles 4ib4_A.span

-parser:protocol relax_wo_clr.xml

-relax:jump_move true

-packing:pack_missing_sidechains false

-mp:scoring:hbond true

-relax:constrain_relax_to_start_coords

## relax_wo_clr.xml XML

<ROSETTASCRIPTS>

<SCOREFXNS>

<ScoreFunction name="frank" weights="franklin2019"/>

</SCOREFXNS>

<TASKOPERATIONS>

<InitializeFromCommandline name="commandline_init"/>

<RestrictToRepacking name="restrict_to_repacking"/>

</TASKOPERATIONS>

<MOVERS>

<AddMembraneMover name="add_memb"/>

<MembranePositionFromTopologyMover name="init_pos"/>

<FastRelax name="fastrelax" disable_design="True" scorefxn="frank" task_operations="commandline_init,restrict_to_repacking" repeats="3"/>

</MOVERS>

<PROTOCOLS>

<Add mover="add_memb"/>

<Add mover="init_pos"/>

<Add mover="fastrelax"/>

</PROTOCOLS>

</ROSETTASCRIPTS>

**Output: 4ib4_A_relax.pdb**

*Lipid*

#1 The SDF file is downloaded from PubChem and conformers are generated with MOE.

**Output: CLR_conformers.sdf**

#2 Generate Rosetta-readable params file for ligand.

ROSETTA/main/source/scripts/python/public/molfile_to_params.py -n CLR -p CLR --conformers-in-one-file CLR_conformers.sdf

**Output: CLR.params, CLR.pdb, CLR_conformers.pdb**

**Docking**

#1 Calculate lipid accessible regions on the protein for global docking using the Rosie server:

- Reduce XYZ coordinates from per atom to center of mass of each residue (centroid) in output PDB
- Change the resname (example: ASP) to water (HOH) in output PDB
- Change the atoms (example: CA) to oxygen (O) in output PDB
- Change ATOM to HETATM in output PDB

**Output: 4ib4_A_positions.pdb**

#2 Prepare docking input file

- Combine **4ib4_A_relax.pdb** with **CLR.pdb**

**Output: 4ib4_A_dock.pdb**

#3 Dock cholesterol to the protein and generate 10 models

## dock.sh command

Rosetta/main/source/bin/rosetta_scripts.linuxgccrelease

-in:file:s 4ib4_A_dock.pdb

-mp:setup:spanfiles 4ib4_A.span

-in:file:extra_res_fa CLR.params

-packing:pack_missing_sidechains false

-parser:protocol dock.xml

-nstruct 10

## dock.xml XML

<ROSETTASCRIPTS>

<SCOREFXNS>

<ScoreFunction name="frank" weights="franklin2019"/>

</SCOREFXNS>

<SCORINGGRIDS ligand_chain="X" width="25" name="lipid">

<LipidMemGrid grid_name="head" weight="0.8" mem_weight="2" ligand_atom="O1" kbpot_file="chol_o_mem_z_smooth_energies"/>

<LipidMemGrid grid_name="tail" weight="1.0" mem_weight="2" ligand_atom="C25" kbpot_file="chol_c25_mem_z_smooth_energies"/>

<ClassicGrid grid_name="classic" weight="0.00000001"/>

</SCORINGGRIDS>

<MOVERS>

<StartFrom name="start" chain="X" >

<PDB filename="4ib4_A_positions" atom_name="O"/>

</StartFrom>

<Transform name="transform" chain="X" box_size="6" move_distance="0.2" angle="20" cycles="500" repeats="5" temperature="5" grid_set="lipid"/>

<MOVERS/>

</ROSETTASCRIPTS>

**Output/**

The following files are included in the **outputs/** directory:

The head and tail atoms of the lipid are optimized independently. The score.sc file contains the individual score terms labeled head_grid_X and tail_grid_X, respectively.

| 4ib4_A.000*.pdb | output files |
| --- | --- |
| score.sc | scorefile |

***Specificity Filter Calculation***

After running the Prediction protocol protein-cholesterol complexes can be further analyzed with the *spec_score* script. *spec_score* is a python script used to predict the likelihood that an integral membrane protein-cholesterol interface is specific.

**Input/**

The following files are included in the **inputs/** directory to run the SpecificityFilter:

| 4ib4_A_pdbs/ | Prediction protocol output pdbs |
| --- | --- |
| buried_area.csv | Names of pockets to investigate with their corresponding buried area (http://schuellerlab.org/dr_sasa/) |
| 4ib4_A_consurf.txt | Consurf rate of evolution predictions (consurf.tau.ac.il) |
| 4ib4_A_netsurfp.txt | Netsurfp lipid accessibility predictions (https://services.healthtech.dtu.dk/service.php?NetSurfP-3.0) |
| 4ib4_A_lips.pdb | Rosetta lipid accessibility prediction (https://rosie.graylab.jhu.edu/mp_lipid_acc) |

#1 Use spec_score command to generate the specificity.csv file

## python spec_score.py

-q inputs/buried_area.csv

-p inputs/4ib4_A_pdbs/

-c inputs/4ib4_A_consurf.txt

-l inputs/4ib4_A_lips.pdb

-n inputs/4ib4_A_netsurfp.txt

**Output/**

The following files are included in the **outputs/** directory:

The output is a csv file containing the Escore, Pscore and specificity score.

| specificity.csv | output file |
| --- | --- |
